# OsNBL3, a mitochondrion‐localized pentatricopeptide repeat protein, is involved in splicing *nad5* intron 4 and its disruption causes lesion mimic phenotype with enhanced resistance to biotic and abiotic stresses

**DOI:** 10.1111/pbi.13659

**Published:** 2021-07-17

**Authors:** Tiancheng Qiu, Xiaosheng Zhao, Huijing Feng, Linlu Qi, Jun Yang, You‐Liang Peng, Wensheng Zhao

**Affiliations:** ^1^ State Key Laboratory of Agrobiotechnology, MOA Key Lab of Pest Monitoring and Green Management Department of Plant Pathology China Agricultural University Beijing China

**Keywords:** OsNBL3, *Oryza sativa*, disease resistance, salt tolerance, pentatricopeptide repeat (PPR) protein, mitochondria, RNA splicing, *nad5*, lesion mimic

## Abstract

Lesion mimic mutants are used to elucidate mechanisms controlling plant responses to pathogen attacks and environmental stresses. Although dozens of genes had been functionally demonstrated to be involved in lesion mimic phenotype in several plant species, the molecular mechanisms underlying the hypersensitive response are largely unknown. Here, a rice (*Oryza sativa*) lesion mimic mutant *natural blight leaf 3* (*nbl3*) was identified from T‐DNA insertion lines. The causative gene, *OsNBL3,* encodes a mitochondrion‐localized pentatricopeptide repeat (PPR) protein. The *nbl3* mutant exhibited spontaneous cell death response and H_2_O_2_ accumulation, and displayed enhanced resistance to the fungal and bacterial pathogens *Magnaporthe oryzae* and *Xanthomonas oryzae* pv. *oryzae*. This resistance was consistent with the up‐regulation of several defence‐related genes; thus, defence responses were induced in *nbl3*. RNA interference lines of *OsNBL3* exhibited enhanced disease resistance similar to that of *nbl3*, while the disease resistance in overexpression lines did not differ from that of the wild type. In addition, *nbl3* displayed improved tolerance to salt, accompanied by up‐regulation of several salt‐associated marker genes. OsNBL3 was found to mainly participate in the splicing of mitochondrial gene *nad5* intron 4. Disruption of *OsNBL3* leads to the reduction in complex I activity, the elevation of alternative respiratory pathways and the destruction of mitochondrial morphology. Overall, the results demonstrated that the PPR protein‐coding gene *OsNBL3* is essential for mitochondrial development and functions, and its disruption causes the lesion mimic phenotype and enhances disease resistance and tolerance to salt in rice.

## Introduction

Programmed cell death (PCD) plays important roles in the embryonic, juvenile and adult phases of plant development. In addition, a type of PCD is often observed during plant response to pathogen attack; this is termed the 'hypersensitive response' (HR). HR is a defence mechanism employed by plants to protect themselves from biotic stress and is usually accompanied by the generation of reactive oxygen species (ROS), expression of pathogenesis‐related (PR) proteins, accumulation of callose and thickening of cell walls at the infection sites (Jones and Dangl, 1996).

Lesion mimic mutants (LMMs), also called *spotted leaf* (*spl*) mutants, spontaneously develop localized cell death lesions resembling those caused by HR in the absence of pathogen infection, abiotic stress or mechanical damage. In many cases, LMMs exhibit significantly enhanced disease resistance; therefore, LMMs are deemed as suitable materials for elucidating the mechanisms underlying plant responses to pathogen attacks and environmental stresses. To date, dozens of genes have been characterized from LMMs and that encode diverse proteins, including porphyrin (Undan *et al*., 2012), transcription factors (Li *et al*., 2004), oxidoreductases (Yang *et al*., 2004), protein kinases (Wang *et al*., 2015a), ubiquitinations (Zeng *et al*., 2004), membrane‐associated proteins (Noutoshi *et al*., 2006), zinc finger proteins (Wang *et al*., 2005), ion channel proteins (Mosher *et al*., 2010), clathrin‐associated adaptor proteins (Qiao *et al*., 2010), nucleotide‐binding site–leucine‐rich repeat (NLR) proteins (Tang *et al*., 2011), mRNA splicing factors (Chen *et al*., 2012), UDP‐*N*‐acetylglucosamine pyrophosphorylase (Wang *et al*., 2015b), AAA‐type ATPase (Fekih *et al*., 2015), eEF1A‐like protein (Wang *et al*., 2017c) and glycine‐rich domain proteins (Zhao *et al*., 2020). The large range of protein types contributes to the characteristics of LMMs, suggesting the molecular mechanisms regulating defence responses in plants are very complicated.

Mitochondria playing a pivotal role during PCD is well reviewed by Huang *et al*., (2016). PCD is closely associated with mitochondrial reactive oxygen species (mtROS) production in plants. mtROS production is often increased during stress and PCD, likely due to mitochondrial electron transport chain (mtETC) inhibition (Huang, *et al*., 2016). mtETC enzymes, such as AOX, protect against PCD (Wu *et al*., 2015). In *Arabidopsi*s, mitochondrial Complex I is responsible for the ROS production that leads to PCD in the *mosaic death 1* (*mod1*) mutant (Wu *et al*., 2015). Mitochondria have also been proposed to be linked with PCD via increased ROS production during pathogen defence. In agreement, a wide variety of mutants in mitochondrial functions have been reported to have altered PCD or pathogen defence phenotypes (Huang, *et al*., 2016). Although plant mitochondrial genomes can encode genes, most of these genes are regulated by proteins encoded by nuclear genes (Schmitzlinneweber and Small, 2008). One of the largest families of proteins that modulate mitochondrial gene expression is pentatricopeptide repeat (PPR) proteins, which can be divided into P and PLS subfamilies according to PPR motifs therein. The P‐subfamily proteins only consist of canonical 35 amino acid‐PPR motifs carrying, whereas the PLS‐subfamily proteins contain short motifs (S; 31 amino acids), long motifs (L; 35–36 amino acids) and E, E+ or DYW domains at the C terminus (Schmitzlinneweber and Small, 2008). The rice genome is predicted to encode 491 PPR proteins (246 P‐subfamily and 245 PLS subfamily). Moreover, 90 E domains and 131 DYW domains have been found in the PLS subfamily (Chen *et al*., 2018). P‐subfamily proteins are mainly involved in mitochondrial RNA splicing, stabilization and translation, while PLS‐subfamily proteins mainly play a role in the C‐U editing of mitochondrial RNA (Barkan and Small, 2014).

Most of the PPR proteins located in mitochondria are involved in modifying the gene expression of subunits of the mtETC, which is composed of respiratory enzyme complexes, such as the nicotinamide adenine dinucleotide (NADH)‐ubiquinone oxidoreductase system (complex I), succinate‐ubiquinone reductase system (complex II), cytochrome b precursor (complex III), cytochrome c oxidase system (complex IV) and ATP synthase system (complex V). Mutations in the PPR genes lead to dysfunction of the mitochondrial ETC, which can cause seed and embryo development defects, growth retardation, pollen abortion, and abiotic stress and ABA sensitivity in plants (Barkan and Small, 2014). In Arabidopsis, the P‐subfamily proteins MTL1 (Haili *et al*., 2016), SLOW GROWTH 3 (Hsieh *et al*., 2015) and OTP43 (De Longevialle *et al*., 2007) have been shown to be localized in mitochondria and are involved in the *cis*‐ or *trans*‐splicing of the introns of genes encoding subunits of complex I. Recently, it was reported that a rice P‐subfamily PPR protein, FLO10, is required for the *trans*‐splicing of the mitochondrial *nad1* intron 1 (Wu *et al*., 2019). Another P‐subfamily PPR protein, RL1 of rice, was specifically involved in the splicing of the mitochondrial *nad4* intron 1 (Wu *et al*., 2020). Several mitochondrion‐localized P‐type PPR proteins have been reported to be involved in the RNA stabilization of genes coding for subunits of complex I. These P‐type PPR proteins include Arabidopsis AtMTFS1 (Haili *et al*., 2013), AtMTFS2 (Wang *et al*., 2017a), PPR19 (Lee *et al*., 2017) and maize PPR78 (Zhang *et al*., 2017). The mitochondrion‐localized PLS‐subfamily PPR proteins, including rice OGR1 (Sungryul *et al*., 2009), MPR25 (Toda *et al*., 2012), Arabidopsis SLO2 (Zhu *et al*., 2012), SLO4 (Weissenberger *et al*., 2017), MEF8 (Diaz *et al*., 2017), maize DEK36 (Wang *et al*., 2017b) and DEK39 (Li *et al*., 2018), are mainly involved in the C‐U editing of genes encoding subunits of complexes I and III. Although these studies shed some light on the roles of PPR proteins in plant growth and development via regulation of mitochondrial RNA metabolism, the functions of most PPR proteins remain unclear.

In the incumbent study, we report *Oryza sativa NBL3* (*OsNBL3*) that encodes a mitochondrion‐localized P‐subfamily PPR protein. The mutation in *OsNBL3* leads to a spontaneous lesion mimic phenotype, concomitant with enhanced disease resistance to *Magnaporthe oryzae* (the causal agent of rice blast disease) and *Xanthomonas oryzae* pv. *oryzae* (*Xoo*; the causative pathogen of rice leaf blight disease) and salt tolerance, and premature leaf senescence. RNA interference (RNAi) lines of *OsNBL3* exhibited enhanced disease resistance similar to that of the *nbl3* mutant (a T‐DNA insertion mutant), while the resistance level in overexpression lines thereof did not differ from that of the wild type. OsNBL3 mainly involves in the *cis*‐splicing of *nad5* intron 4 and thus contributes to the precise assembly and construction of mitochondria.

## Results

### Phenotypic characterization of the *nbl3* mutant

**Figure 1:**
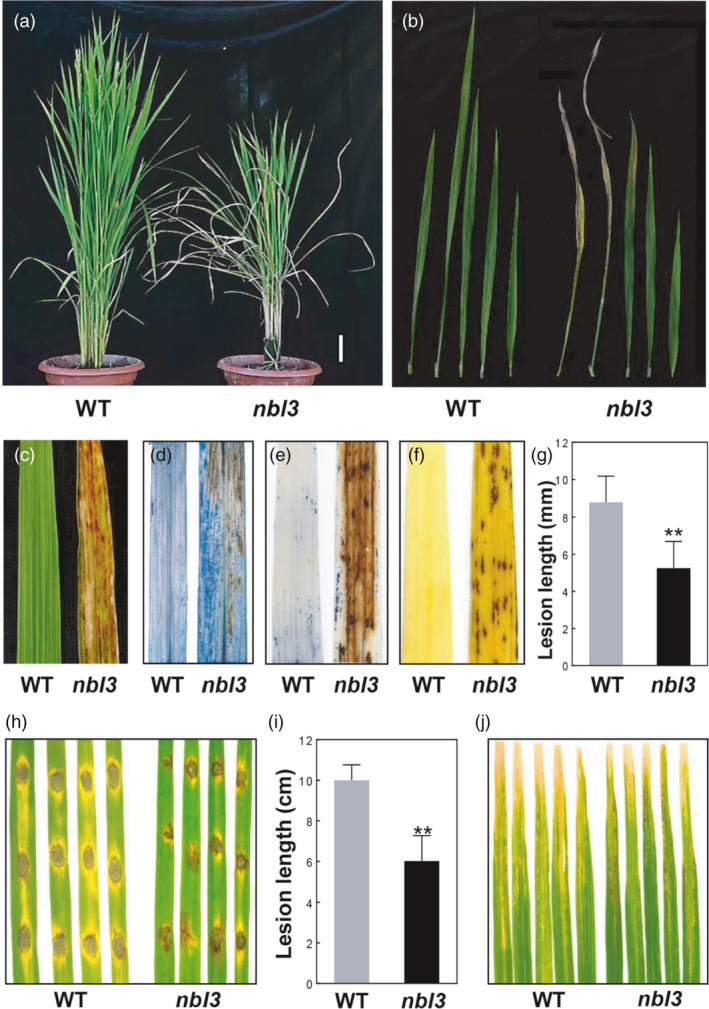
The *nbl3* mutant exhibits lesion mimic phenotype companied with reactive oxygen species burst, accelerated leaf senescence and enhanced disease resistance. (a) Whole plants of the wild type (WT) and the *nbl3* mutant at flowering stage in the paddy field. Scale bars = 10.0 cm. (b) Lesion mimic phenotypes on different leaves of the *nbl3* mutant at the pollination stage, compared with that of wild type. Leaves from lower to upper were arrayed from right to left, respectively. (c) The second leaf of the wild type and the *nbl3* mutant. (d–f) Photographs of leaves stained with Trypan blue, NBT and DAB. (g) Lesion length in leaves of (h) indicates significant differences between WT and *nbl3* leaves. Data were shown as means ± SD, *n* = 10 (**P < 0.01; Student’s *t*‐test). (h) The lesions on the WT and *nbl3* leaves at 96‐hour post‐inoculation with isolate H535 of *M. oryzae*. (i) Lesion length in leaves of (j) indicates significant differences between the WT and *nbl3* leaves. Data were shown as means ± SD, *n* = 15 (**P < 0.01; Student’s *t*‐test). (j) The lesions on the WT and *nbl3* leaves after two‐week post‐inoculation with *Xoo* strain *PXO*99.

The rice mutant *nbl3* (*natural blight leaf 3*) was identified in the paddy field growing T‐DNA insertion lines of Geng (japonica) rice cv. Aichiasahi. Under field conditions (Beijing), the lower leaves of *nbl3* mutants exhibited irregular, brown necrosis lesions at their tips from approximately 30 days after germination. The lesions then expanded from the tip to the whole leaf, leading to severe leaf withering and premature senescence (Figure 1a). The cell death lesions appeared on the *nbl3* plants acropetally (Figure 1b), and by the heading stage, the *nbl3* plants exhibited a typical senescence phenotype (Figure 1c). Under greenhouse conditions, the mutant phenotype of *nbl3* was delayed and was less severe than its field phenotype. After Trypan blue staining, the *nbl3* mutant exhibited dark blue spots, indicating the emergence of cell death or membrane damage on the leaves (Figure 1d). Reactive oxygen species (ROS; e.g. H_2_O_2_) accumulation has been reported in many LMMs (Chen *et al*., 2012; Ma *et al*., 2019; Qiao *et al*., 2010; Wang *et al*., 2017c; Zhao *et al*., 2020). When tetranitroblue tetrazolium chloride (NBT) solution and 3,3′‐diaminobenzidine (DAB) staining were used to assess ROS in the *nbl3* mutant, intense brown staining accentuated surrounding lesion sites on the leaves, as opposed to in the wild‐type leaves (Figure 1e,f). This indicates that H_2_O_2_ accumulation occurred in the *nbl3* leaves. In addition to cell death and premature senescence, several agronomic traits including plant height, tiller number and 1000‐grain weight were affected in the *nbl3* plants (Figure S1).

### Enhanced resistance of *nbl3* against *M*. *oryzae* and *Xoo*, accompanied by constitutive expression of defence‐related genes

The appearance of spontaneous leaf spot is often concomitant with enhanced disease resistance. To determine whether the *nbl3* mutation led to enhanced resistance to rice pathogens, one‐month‐old seedlings of the rice cultivar Aichiasahi (wild type) and the *nbl3* mutant were inoculated with *M. oryzae*. The *nbl3* plants growing in greenhouse conditions did not display lesions when inoculated with H535, a virulent isolate of *M*. *oryzae*, using the punch inoculation method. The size of lesions on the *nbl3* leaves was significantly smaller than that of lesions on the wild‐type leaves at 96 h post‐inoculation (hpi; Figure 1g,h). Two‐month‐old seedlings were also inoculated with the bacterial blight pathogen *Xoo* strain PXO99. It was found that the lesions on the *nbl3* leaves were much smaller than those on the wild‐type plants two weeks post‐inoculation (Figure 1i,j). These results demonstrate that the *nbl3* plants display significantly enhanced resistance to both *M*. *oryzae* and *Xoo*.

**Figure 2:**
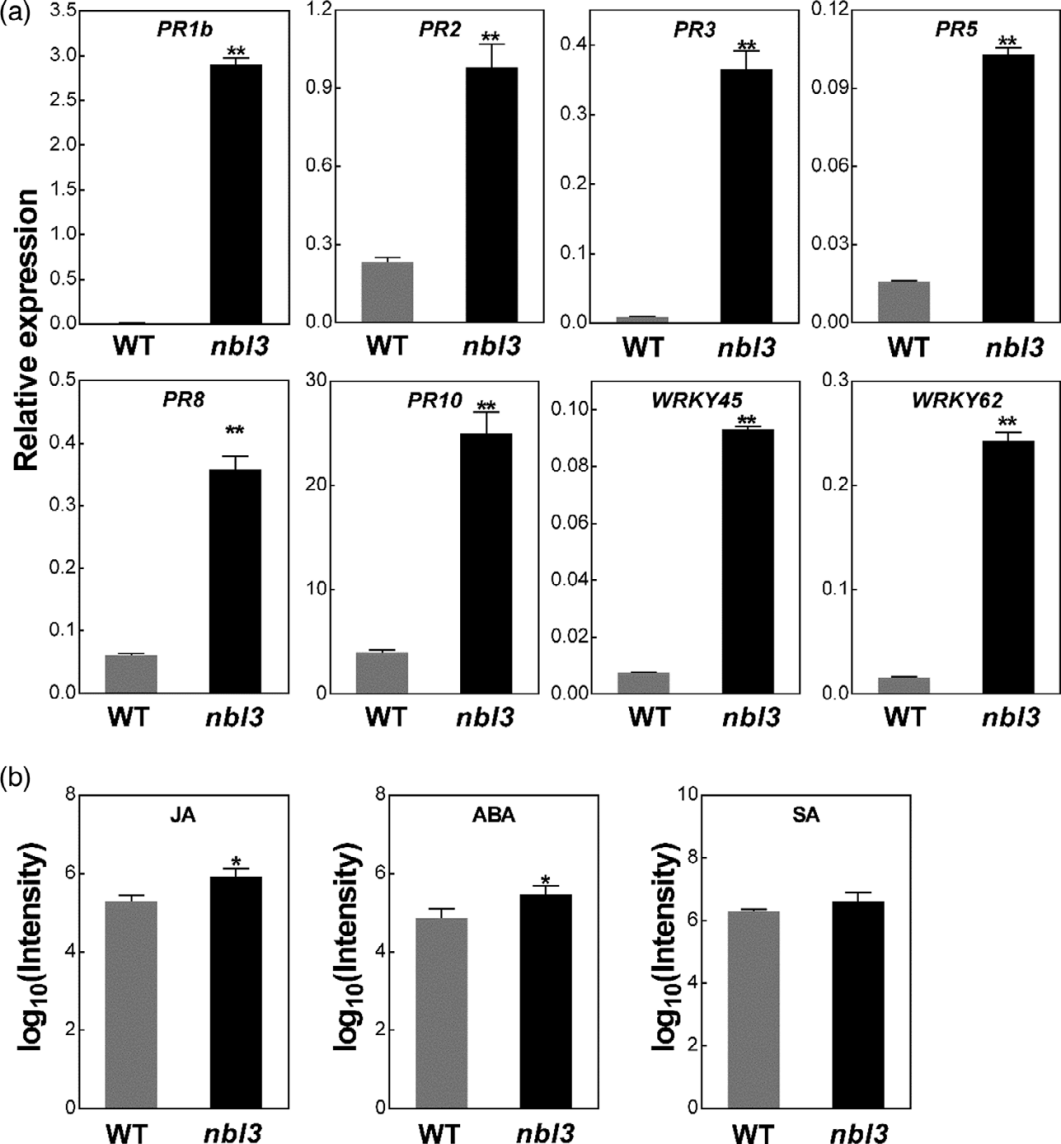
Constitutive expression of several defence‐related genes and intensity of three phytohormones in the *nbl3* mutant and wild type. (a) Pathogenesis‐related and defence‐related genes in the wild type (WT) and *nbl3* were analysed by RT‐qPCR at the four‐leaf stage (before the appearance of visible lesions in the *nbl3*). The results were obtained in three independent biological replicates. Pathogenesis‐related genes include *OsPR1b*(*LOC_Os07g03710*), *OsPR2*(*LOC_Os01g71340*), *OsPR3*(*LOC_Os10g39680*), *OsPR5*(*LOC_Os12g43380*), *OsPR8*(*LOC_Os10g28080*) and *OsPR10*(*LOC_Os12g36880*). Defence‐related genes include *OsWRKY45*(*LOC_Os05g25770*) and *OsWRKY62*(*LOC_Os09g25070*). Rice gene *OsActin* (*LOC_Os03g50885*) was used as an internal control. Data were shown as means ± SD, *n* = 3 (**P < 0.01; Student’s *t*‐test). (b) Histogram showing the log_10_ intensity of Salicylic acid (SA), jasmonic acid (JA) and abscisic acid (ABA) in wild type and *nbl3*. Two‐month‐old seedlings grown in the paddy field were used to extract SA, JA and ABA. Log_10_ intensity values were shown as means ± SD, *n* = 3 (*P < 0.05; Student’s *t*‐test).

In many rice LMMs, the constitutive expression of defence‐response genes has been found to be accompanied by lesion development (Ma *et al*., 2019; Zhao *et al*., 2020). To determine whether the transcription of defence‐related genes was affected in the *nbl3* mutant, expression analyses were performed using RT‐qPCR. Several pathogenesis‐related protein genes, including *OsPR1b*, *OsPR2*, *OsPR3*, *OsPR5*, *OsPR8* and *OsPR10*, and two defence‐related genes, *OsWRKY45* and *OsWRKY62,* were significantly up‐regulated in *nbl3* (Figure 2a). These results are consistent with the enhanced disease resistance of *nbl3*. Among these up‐regulated genes, *OsWRKY62* is known to be involved in the jasmonic acid (JA) signalling pathway (Liu *et al*., 2016), and the other genes are well known to be involved in the salicylic acid (SA) signalling pathway (Tang *et al*., 2011; Wang *et al*., 2019). Furthermore, the intensity of jasmonic acid, abscisic acid and salicylic acid are higher in *nbl3* than that in the wild type (Figure 2b). These results suggest that the mutation in *nbl3* confers enhanced disease resistance and other phenotypes, possibly mediated by integrated signalling pathways.

### Cloning of *OsNBL3* and its expression patterns

Genetic analysis demonstrated that the *nbl3* mutation is a recessive trait that is co‐segregated with the T‐DNA insertion (Table S1). Thereafter, SiteFinding TAIL‐PCR procedures were used to isolate genomic sequences flanking both ends of the T‐DNA. The results showed that the T‐DNA was integrated into chromosome 3. Mapping the sequences onto the *O. sativa* cv. Nipponbare genome (The Rice Genome Annotation Project; http://rice.plantbiology.msu.edu/) revealed that the insertion site was located in the 3′‐untranslated region of an annotated gene *LOC_Os03g06370* (Figure S2a). This insertion event was confirmed by specific PCR using two pairs of primers flanking the insertion site (T‐DNA borders; Figure S2b). Using the two pairs of primers to perform the semi‐quantitative RT‐PCR analysis, the transcript of *LOC_Os03g06370* was not detectable in the *nbl3* seedlings (Figure S2c). The RT‐qPCR analysis also confirmed that the expression level of *LOC_Os03g06370* was dramatically lower in *nbl3* than in the wild type (Figure S2d). These results indicated that the mutation in *LOC_Os03g06370* was responsible for the *nbl3* phenotypes. This was further confirmed by overexpression and RNAi analyses of the gene using transgenic procedures (see below). Thus, the *LOC_Os03g06370* gene is renamed as *OsNBL3* in this study.

**Figure 3:**
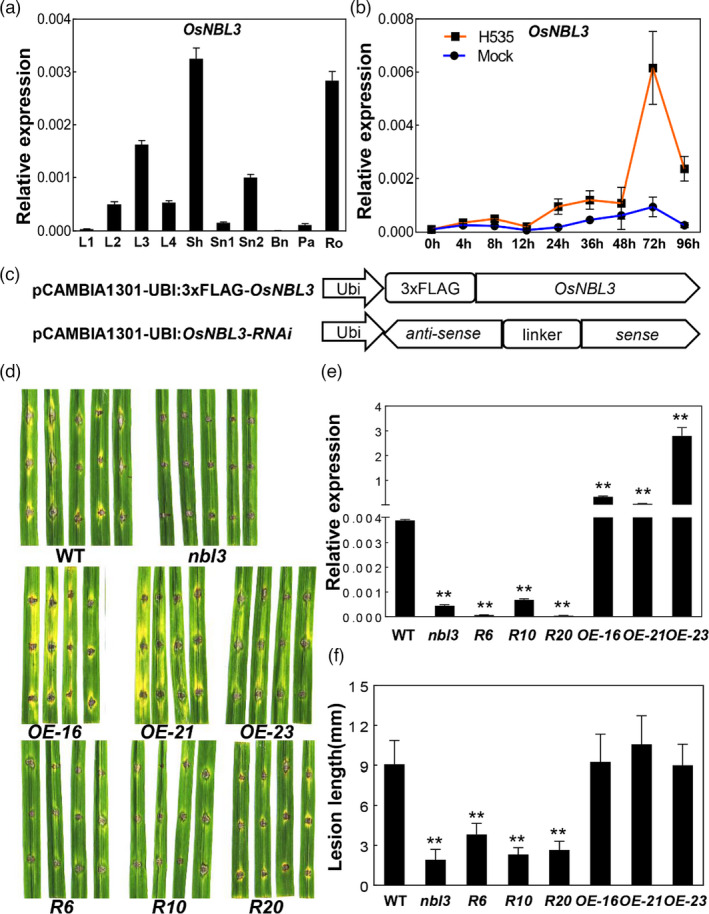
Expression patterns of *OsNBL3* and enhanced disease resistance of RNA interfere plants. (a) RT‐qPCR analysis of *OsNBL3* expression patterns in different tissues of wild‐type plants. Flag leaf (L1), second leaf (L2), third leaf (L3), fourth leaf (L4), Sheath (Sh), Stem node 1(Sn1), Stem node 2(Sn2), Basal node(Bn), panicle(Pa), Root(Ro). Data were shown as means ± SD, *n* = 3. Rice *OsActin* (*LOC_Os03g50885*) gene was used as an internal control. (b) RT‐qPCR analysis of *OsNBL3* expression level at different time after inoculation with the compatible isolate H535 of *M. oryzae*. Two‐week‐old seedlings were used for inoculation. The seedlings sprayed only with 0.025% Tween 20 were used as negative control (Mock). Rice *OsActin* (*LOC_Os03g50885*) gene was used as an internal control. Data were shown as means ± SD, *n* = 3, (**P < 0.01; Student’s *t*‐test). (c) Schematic diagrams of the *OsNBL3‐OE* and RNAi structures. Ubi, Maize ubiquitin promoter; 3xFlag, three tandem repeat Flag tag; a cDNA fragment from 238 bp to 797 bp downstream of *OsNBL3*’s ATG was used sense and anti‐sense sequence. (d) The lesion on the wild type (WT) and *nbl3* leaves at 96‐hour post‐inoculation with H535. (e) Expression analysis of *OsNBL3* in the wild type(WT) and OsNBL3‐OE or OsNBL3‐RNAi lines by RT‐qPCR, respectively. Data were shown as means ± SD, *n* = 3 (**P < 0.01; Student’s *t*‐test). OsNBL3‐RNAi lines include R6, R10, R20; *OsNBL3‐OE* lines include OE‐16, OE‐21, OE‐23. (f) Lesion length in leaves (b) of the wild type, *nbl3*, OsNBL3‐OE and OsNBL3‐RNAi lines (**P < 0.01; Student’s *t*‐test). Data were shown as means ± SD, *n* = 10.

Phylogenetic analysis showed that *OsNBL3* shared similarity with its homologues in both monocot and dicot plants, and had especially high level of identity with homologues in grasses (Figure S2e). To determine the expression patterns of *OsNBL3* in rice, RT‐qPCR analyses were performed. It was revealed that *OsNBL3* was constitutively expressed in all tested tissues, with relatively higher expression in the sheath and roots, and lower expression in the basal node and flag leaves at the heading stage (Figure 3a). In addition, the expression level of *OsNBL3* in seedlings was induced by inoculation with the *M*. *oryzae* isolate H535; the induced expression reached a peak at 72 hpi (Figure 3b).

### RNAi plants also exhibited enhanced disease resistance

To further confirm that the disruption of *OsNBL3* was responsible for the *nbl3* phenotypes, we firstly tried to generate knockout lines of *OsNBL3* using the CRISPR/Cas9 method (Shan *et al*., 2013). However, we were unable to obtain transgenic plants. It is possible that the knockout of *OsNBL3* is lethal. Therefore, OsNBL3‐RNAi transgenic lines were generated (Figure 3c), and the transcript levels of *OsNBL3* in each homozygous line were confirmed by RT‐qPCR analysis (Figure 3e). Exceeding expectations, the RNAi plants had shorter plant heights similar to those of the *nbl3* mutant, while no clear spontaneous cell death was observed on the leaves of the RNAi plants (Figure S3a). The intact mRNA level was detected by RT‐PCR analysis using RT‐F and RT‐R as primers; the result showed that the transcripts of *OsNBL3* in the mutant are lower than that in the RNAi plants (Figure S3b), which may be attributed to the phenotypic difference. To determine whether the RNAi plants exhibited enhanced disease resistance, the resistance of three independent RNAi lines to *M*. *oryzae* was tested using the punch inoculation method. The results showed that all the tested lines displayed enhanced resistance (Figure 3d,f). In addition, expression analysis showed that two selected defence‐related marker genes, *OsPR1b* and *OsPR5,* were significantly up‐regulated in the RNAi lines (Figure S4). Overexpression (OE) lines of the *OsNBL3* gene were also generated (Figure 3c). In contrast to the RNAi lines, the OE lines did not exhibit obvious reduced or enhanced resistance to *M. oryzae* compared with the wild type (Figure 3d,f). These results further demonstrate that disruption of *OsNBL3* is responsible for the enhanced disease resistance.

### Both the *nbl3* mutant and RNAi plants exhibit enhanced salt tolerance

**Figure 4:**
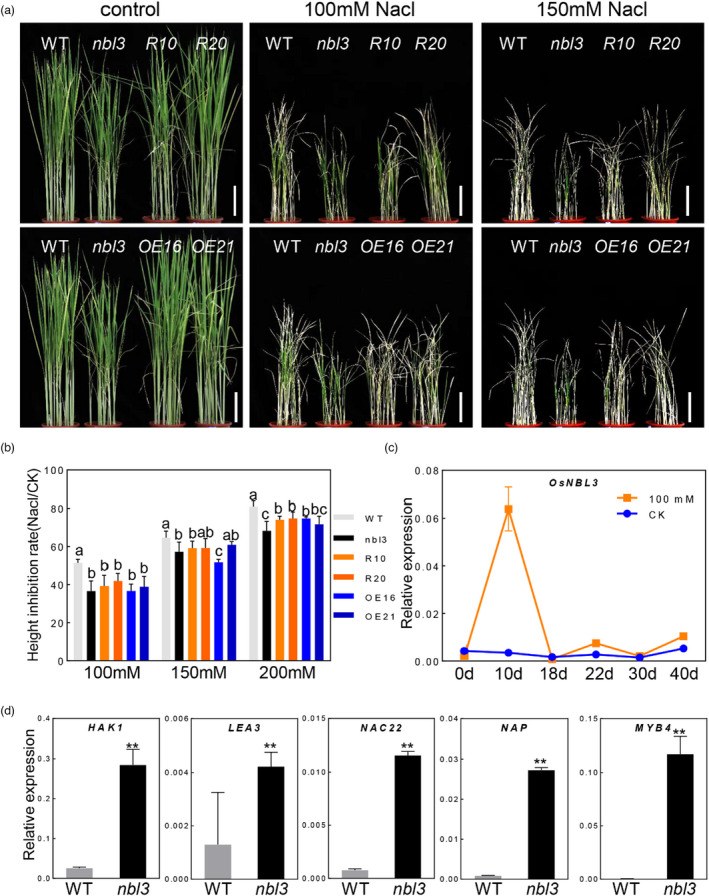
Disruption of *OsNBL3* leads to enhanced salt tolerance. (a) Morphology of 40‐day‐old seedlings of the wild type, *nbl3*, RNAi‐10, ‐20 and OE‐16, ‐21 that all were continuously irrigated with 100, 150 mm NaCl or water only, for thirty days. Scale Bar = 10 cm. (b) The height inhibition rate of seven‐day old seedlings grown on agar plates containing 100, 150 and 200 mm NaCl, respectively. Data were shown as means ± SD, *n* = 5, (Values with same superscript letters are of no significant difference (*P* > 0.05), those with different letters are of significant or extreme difference (*P* < 0.05)). (c) RT‐qPCR analysis of temporal expression patterns of *OsNBL3* in the wild‐type seedlings that were continuously irrigated with 100 mm NaCl. The *OsActin* gene (*LOC_Os03g50885*) was used as an internal control. Data were shown as means ± SD, *n* = 3, (**P < 0.01; Student’s *t*‐test). (d) RT‐qPCR analyses of constitutive expression of several the salt tolerance‐related genes in seedlings of the *nbl3* and wild type. The *OsActin* gene (*LOC_Os03g50885*) was used as an internal control. Data were shown as means ± SD, *n* = 3, (**P < 0.01; Student’s *t*‐test).

It has been reported that the mechanism underlying rice LMMs is regulated by hormones and abiotic stresses (Mosher *et al*., 2010; Wang *et al*., 2015a; Yamanouchi *et al*., 2002). To evaluate whether *OsNBL3* is associated with abiotic stresses, 10‐day‐old seedlings, grown in normal conditions, were continuously irrigated with 100 mm and 150 mm NaCl for thirty days, respectively. Compared with the wild‐type plants, a greater number of *nbl3* and two RNAi plants survived at both salt concentrations, while the two OE lines showed fewer survived plants (Figure 4a), which indicates that *nbl3* and RNAi plants are more tolerant to salt. The germinated seeds were placed on agar plates containing 0, 100, 150 and 200 mm NaCl, and the plant height and fresh weight were measured after seven days. Compared with the wild‐type plants, all the *nbl3*, RNAi and OE plants showed lower inhibition rate of both plant height and fresh weight, at each salt concentration (Figure 4b and Figure S5a,b), further indicating the involvement of *OsNBL3* in salt responses. The root length of both the *nbl3* and wild‐type seedlings grown on agar plates containing 100 mm NaCl were also measured from two to five days post‐planting. The inhibition rate of root growth was significantly lower for *nbl3* than for the wild type at each sampling point (Figure S5c–e). RT‐qPCR analysis was then conducted to determine the expression of *OsNBL3* in response to salt treatment. The result showed that *OsNBL3* expression was induced by NaCl with a peak at 10 days post‐irrigation (Figure 4c). In addition, the expression analysis showed that the potassium transport gene *OsHAK1*, the late embryogenesis abundant protein‐encoding gene *OsLEA3* and the transcription factor genes *OsNAC22*, *OsNAP* and *OsMYB4*, which are salt tolerance‐related genes (Chen *et al*., 2015; Chen *et al*., 2014; Hong et al., 2016; Hu, 2008; Vannini *et al*., 2006), were significantly up‐regulated in the *nbl3* plants compared with in the wild type (Figure 4d). These results demonstrate that *OsNBL3* contributes in response to salt stress, and disruption of the gene results in enhanced salt tolerance.

### OsNBL3 is a mitochondrion‐localized P‐type PPR protein

**Figure 5:**
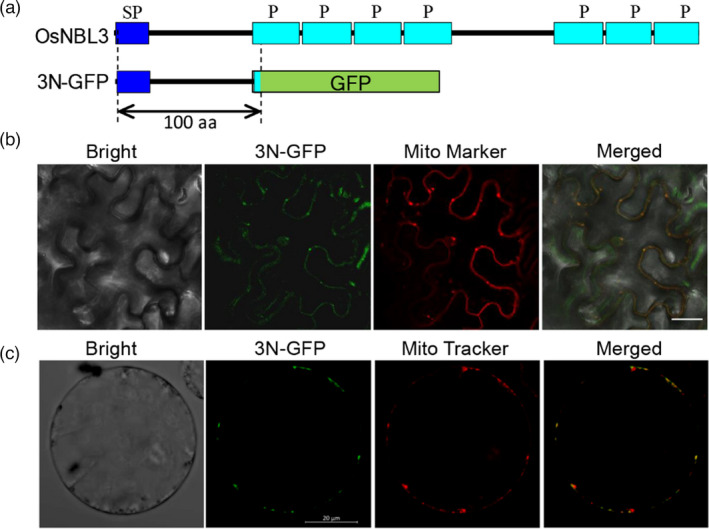
Subcellular localization of OsNBL3. (a) Schematic diagrams of OsNBL3 and 3N‐GFP structure, in which the N terminus of OsNBL3 was fused in frame with GFP. Predicted mitochondrion signal peptide (SP) and PPR repeats (P) by https://ppr.plantenergy.uwa.edu.au/ were indicated. (b) Confocal microscopic images showing co‐localization of the 3N‐GFP and pCXSN‐COX4‐RFP, in which the Mito marker COX4 was fused with red fluorescent protein (RFP), Bar = 25 μm. (c) Confocal microscopic images showing co‐localization of the 3N‐GFP and a Mito tracker in rice protoplasts, Bar = 20 μm.

OsNBL3 encodes a protein with 409 amino acid residues. According to the prediction by https://ppr.plantenergy.uwa.edu.au/ (Cheng *et al*., 2016), OsNBL3 is a PPR protein that harbours seven canonical P‐type PPR repeats (Figure 5a). TargetP prediction analysis showed that the OsNBL3 protein has a mitochondrion‐targeting signal at its N terminus. To determine the subcellular location of OsNBL3, the OsNBL3‐GFP construct was made in which the full‐length cDNA was fused with GFP. When the OsNBL3‐GFP construct was transformed into the epidermal cells of the *N. benthamiana* leaf, no GFP signals were observed. It is possible that the entire OsNBL3‐GFP protein was difficult to express or was easily degraded. Therefore, another construct (3N‐GFP) was made in which the GFP was fused to the N terminus of OsNBL3, harbouring the putative mitochondrion‐targeting signal (Figure 5a). When 3N‐GFP was transiently expressed in the epidermal cells of the *N. benthamiana* leaf, the green fluorescent signals of 3N‐GFP overlapped with signals from the mitochondria Mito‐Marker (Figure 5b). When 3N‐GFP was transiently expressed in rice protoplasts, similar overlays between GFP and signals from the mitochondria dye, Mito‐Tracker Red, were also observed (Figure 5c). These results indicate that OsNBL3 is a mitochondrion‐localized protein.

### OsNBL3 participates predominantly in the splicing of *nad5* intron 4

**Figure 6:**
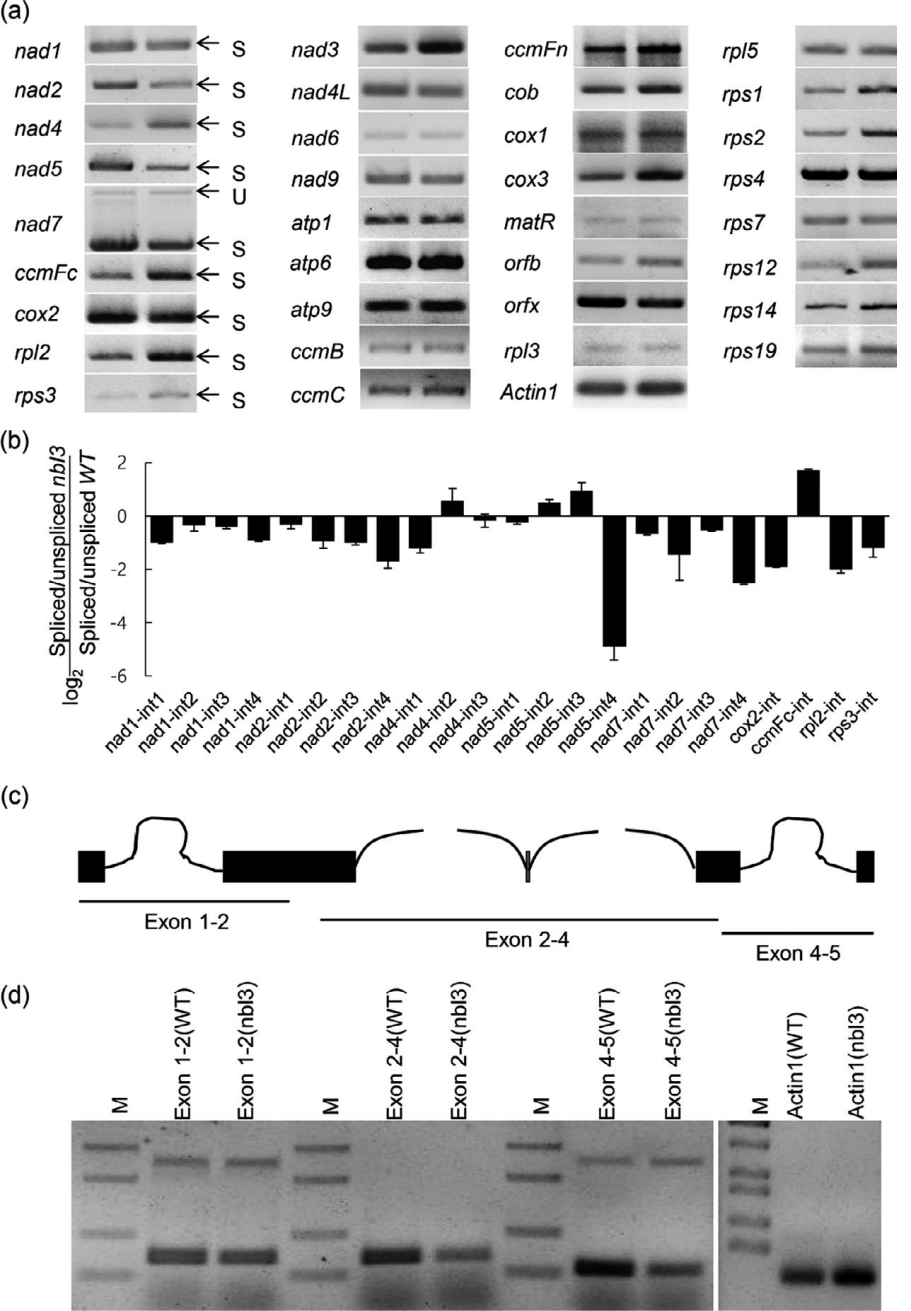
OsNBL3 participates in splicing of *nad5* intron 4. (a) Semi‐quantitative RT‐PCR analyses of all rice mitochondria intron‐containing genes and other mitochondria genes in the wild type and *nbl3* mutant. U and S indicate unspliced and spliced transcripts, respectively. (b) RT‐qPCR analyses of all 23 group II introns in mitochondria. Histogram showing the log2 ratio of spliced to unspliced RNA in the *nbl3* compared with the corresponding value for the wild type. The results are from three biological replicates; data were shown as means ± SD, *n* = 3. (c) Schematic representation of the rice mitochondrial *nad5* gene. Black boxes indicate exons; black curve lines indicate introns, (d) Semi‐quantitative RT‐PCR analysis of four *nad5* intron splicing events in the wild type and *nbl3* mutant. The rice *Actin1* gene (*LOC_Os07g38730*) was used as an internal control and guided amplification for 25 cycles.

It had previously been implicated that P‐type PPR proteins participate in plant organelle RNA metabolism, including 5′ processing, intron splicing and translation (Barkan and Small, 2014). Given that OsNBL3 is a mitochondrion‐targeting protein, CR‐RT‐PCR procedures were firstly used to examine whether the 5′ processing of respiratory complex genes was affected in the *nbl3* mutant. Among 17 analysed genes, the amplified products of each gene in the wild‐type and *nbl3* plants were the same size (Figure S6) suggesting that 5′ processing was not affected by the mutation in *nbl3*. Next, the mature transcripts of 34 mitochondrial genes were examined using total RNA from 30‐day‐old seedlings of the wild‐type and *nbl3* plants with specific primers (Figure 6a). The results showed that only the mature *nad5* transcripts were conspicuously reduced in *nbl3*.

There are four introns in the *nad5* gene, including two *cis*‐introns and two *trans*‐introns (Bonen, 2008). To determine whether the reduction in the *nad5* transcripts resulted from splicing defects in *nbl3*, RT‐qPCR was conducted to analyse the ratio of spliced to unspliced transcripts of all the 23 introns in mitochondria. The splicing efficiency of *nad5* intron 4 was dramatically reduced in *nbl3* compared with in the wild type (Figure 6b). To further verify improper splicing of *nad5* in *nbl3*, reverse transcription PCR was performed, allowing amplification across adjacent exons and detection of each splicing event in *nad5* (Figure 6c). The amount of *cis*‐spliced transcripts of exons 4–5 (exon 4 + exon 5) was significantly reduced in the *nbl3* mutant (Figure 6d). These results suggest that OsNBL3 is required for the *cis*‐splicing of the mitochondrial *nad5* intron 4. Interestingly, the amount of *trans*‐spliced transcripts of exons 2–4 (exon 2 + exon 3 + exon 4) was also weakly reduced in the *nbl3* mutant, suggesting that OsNBL3 also participates in the *trans*‐splicing of *nad5* in mitochondria.

### *OsNBL3* mutation affects NADH dehydrogenase activity and mitochondrial morphology

**Figure 7:**
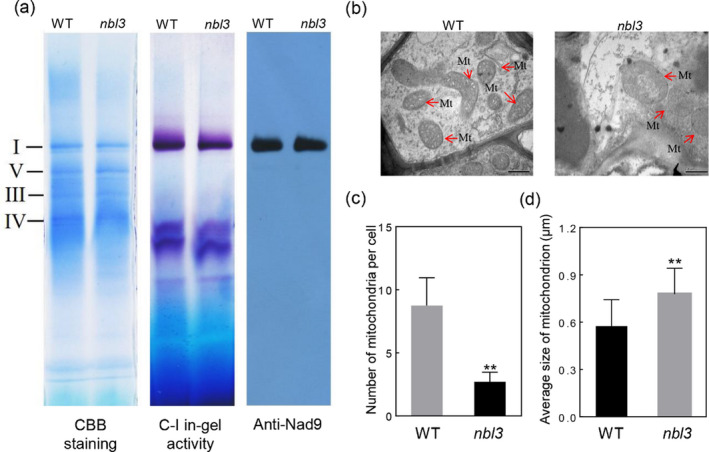
*OsNBL3* mutation affects NADH dehydrogenase activity and mitochondrial morphology. (a) Left, Coomassie Brilliant Blue (CBB) staining of mitochondrial proteins from the wild type (WT) and the *nbl3* mutant by Blue native‐polyacrylamide gel electrophoresis (BN–PAGE). Middle, in‐gel NADH dehydrogenase activity staining for Complex I, C‐I: complex I. Right, Western blot analysis with antibody against Nad9 (a subunit of complex I). (b) Transmission electron microscope images of mitochondria in wild‐type (WT) and *nbl3* leaves. The red arrows indicate mitochondria (Mt). Bar = 0.5 μm. (c) Number of mitochondria per cell in the mutant *nbl3* and WT. (d) Average size of mitochondrion of the mutant *nbl3* and WT.

NAD5 is a subunit of NADH dehydrogenase (complex I) in the mitochondrial respiratory chain. To investigate whether complex I and mitochondrial morphology were affected by the disruption of *OsNBL3*, the assembly and function of the mitochondrial respiratory chain and complex I in‐gel activity assays were conducted by Blue Native‐PAGE (BN‐PAGE) and Coomassie Brilliant Blue (CBB) staining, respectively. No significant difference in the assembly of complex I in *nbl3* was observed in BN‐PAGE as compared with the wild type, and Western blot using an antibody against NAD9 (a subunit of complex I) showed little reduction in *nbl3*. However, the activity of complex I was reduced in *nbl3* compared with the wild type (Figure 7a). Transmission electron microscopy assays were used to observe the ultrastructure of the mitochondria in mesophyll cells from eight‐week‐old *nbl3* and wild‐type plant leaves. The overall number of mitochondria was lower in *nbl3* than in the wild type, and the overall size of mitochondria was relatively greater in *nbl3*. Specifically, swelling cristae with vesicle‐like structures and reduced intermembrane content were observed in the *nbl3* mesophyll cells (Figure 7b and Figure S7a).

Disruption of ETC in plant mitochondria usually leads to the induction of alternative respiratory pathway genes that are known to be mitochondrial stress markers (Vanlerberghe, 2013). RT‐qPCR analysis showed that there were significantly more *AOX1a* and *AOX1c* transcripts in the leaves of 30‐day‐old *nbl3* seedlings than in the leaves of wild‐type seedlings (Figure S7b). Plant mitochondria have internal and external NADH dehydrogenases that function as alternative dehydrogenases (Rasmusson and Wallstrom, 2010). Expression analysis showed that the internal NADH dehydrogenase genes, *NDB2* and *NDB3*, and the external NADH dehydrogenase genes, *NDA1* and *NDC1,* were expressed at a higher level in the leaves of four‐week‐old *nbl3* seedlings than in four‐week‐old wild‐type seedlings (Figure S7b). These results demonstrate that loss of *OsNBL3* function affects the assembly of complex I, leading to the induction of the alternative respiratory pathway.

## Discussion

### The *nbl3* is a LMM mutant caused by the disruption of a PPR gene

Plant cell death and structural adaptations may protect plants from biotic and abiotic stresses, including pathogens, salt and high temperature but ROS (Ma *et al*., 2019; Yamanouchi *et al*., 2002; Zeng *et al*., 2004). The molecular mechanism underlying plant cell death and defence responses in LMMs mutants have been partially elucidated. LMM mutants show spontaneous HR‐like necrotic lesions and bursts of ROS, and activate the expression of defence genes (Wang *et al*., 2017c; Xu *et al*., 2014). The *nbl3* mutant identified in the study exhibited growth retardation, leaf wilting and premature senescence (Figure 1a–c). Interestingly, unlike most identified LMM mutants that usually display regular‐shaped reddish‐brown spots, although some of them are regulated developmentally or environmentally. In the case of *nbl3*, the typical lesion mimic spots of *nbl3* only occurred at the seedling stage. From the tilling stage, the *nbl3* does not exhibit obvious dark spots, while the leaves of *nbl3* gradually withered from the lower to upper leaves. Histochemical staining using Trypan blue, NBT and DAB showed that the *nbl3* mutant displayed ROS accumulation and cell death in the leaves (Figure 1d–f). These symptoms have also been observed in other LMM mutants (Wang *et al*., 2017c; Zhao *et al*., 2020). These results indicated that *nbl3* is a new LMM mutant.

In addition to ROS bursts and cell death, many LMM mutants exhibit enhanced disease resistance (Chen *et al*., 2012; Ma *et al*., 2019; Qiao *et al*., 2010; Yamanouchi *et al*., 2002). In the present study, it was found that the *nbl3* mutant showed enhanced resistance against both fungal and bacterial pathogens (Figure 1g–j). This suggests that OsNBL3 may play roles in regulating PCD and resistance signalling pathways in plants (Figure 2, 3b). Interestingly, the RNAi plants did not show spontaneous cell death in the same way that the *nbl3* mutant did (Figure S3a). However, the RNAi plants did display enhanced resistance to *M. oryzae* (Figure 3d,f). An explanation for this could be that the transcripts were destroyed in the *nbl3* mutant by a T‐DNA insertion within the 3′‐untranslated region of *OsNBL3*. Meanwhile, the down‐regulation of *OsNBL3* in the RNAi plants was mediated via a post‐transcriptional silencing mechanism, which allowed for a few intact transcripts to be present. Consistent with the explanation, the transcripts of *OsNBL3* in the RNAi plants were more than that of mutants, but less than that of wild type (Figure S3b).

*OsNBL3* encodes a mitochondrion‐localized PPR protein (Figure 5). As RNA binding factors, PPR proteins regulate the RNA expression in organelles, thereby affecting plant growth and development. There are many reports involving PPR mutants that have shown that PPR proteins are involved in regulating the physiological functions of plant growth and development. Such PPR mutants have displayed cytoplasmic male sterility (CMS) (Hu *et al*., 2012; Huang *et al*., 2015), defective seed or embryo development (Liu *et al*., 2020; Wang *et al*., 2017b), restricted plant growth (Xie *et al*., 2016; Zhu *et al*., 2012), organelle development defects (Lin *et al*., 2015; Wang *et al*., 2018b), albino leaf phenotype (Su *et al*., 2012; Tang *et al*., 2017) and insensitivity to abiotic stress (Yuan and Liu, 2012; Zsigmond *et al*., 2012). In this study, *nbl3* showed almost all of the physiological phenotypes of PPR mutants, such as growth retardation (Figure 1a), reduced seed setting rate (Figure S1) and enhanced salt tolerance (Figure 4). However, unlike the other PPR mutants, disruption of a mitochondrion‐localized PPR gene causes the *nbl3* phenotype with enhanced disease resistance.

### OsNBL3 is involved in splicing *nad5* introns

PPR proteins are usually encoded by nuclear genes and then transported to organelles to regulate RNA metabolism, including intron splicing, RNA editing, 5′‐ and 3′‐modification, RNA degradation and translation and other post‐transcriptional modification processes (Barkan and Small, 2014). It is well known that mitochondria provide energy for cell processes. Group II introns are ubiquitous in the organelle genomes of flowering plants. There are 23 group II introns in rice mitochondria, 19 of which are distributed in the genes encoding subunits of the NADH dehydrogenase complex (complex I) (Bonen, 2008). Complex I is the first enzyme complex in the mitochondrial respiratory chain, the starting point for electrons to enter the respiratory chain, and essential for mitochondrial energy production and electron transfer (Dai *et al*., 2018; Wu *et al*., 2019). It has been reported that defects in the intron splicing of *nad* mRNAs could lead to partial or complete reductions in the activity of ETC complex I, and disturb plant growth and development. Recently, several P‐type PPR proteins have been identified as splicing factors of *nad* in Arabidopsis and maize (Colas des Francs‐Small *et al*., 2014; Zhang *et al*., 2017). Defects in *nad* gene intron splicing result in a significant decrease in the assembly and activity of complex I and overexpression of AOX genes in the *misf* and *ppr19* mutants of *Arabidopsis thaliana* (Lee *et al*., 2017; Wang *et al*., 2018a). The maize PPR gene mutants *dek37*, *emp12* and *ppr20* displayed compromised splicing efficiencies of *nad2* introns, and mitochondrial morphology and seed development were also affected (Dai *et al*., 2018; Sun *et al*., 2019; Yang *et al*., 2020). Furthermore, the other two PPR proteins DEK41 and EMP602 have been shown to be required for the splicing of *nad4* introns and seed development (Ren *et al*., 2019; Zhu *et al*., 2019). The PPR protein ZmSMK9 affects the development of the kernel and plant architecture by participating in the splicing of *nad5* introns in maize (Pan et al., 2019). In rice, two PPR proteins FLO10 and RL1 were reported to be involved in the splicing of *nad1* intron 1 and *nad4* intron 1, respectively (Wu *et al*., 2019; Wu *et al*., 2020). n*ad5* contains four group II introns, of which introns 1 and 4 are *cis*‐spliced, while introns 2 and 3 are *trans*‐spliced (Bonen, 2008). In the present study, it was found that the disruption of the PPR gene *OsNBL3* compromised the splicing of *nad5* introns in mitochondria (Figure 6b–d). As mention above, RNA metabolism in organelles is essential for their functions. It was considered that P‐type PPR proteins are associated with RNA splicing. In rice genome, there are 246 genes encoding P‐type PPR proteins (Chen *et al*., 2018). Among them, only few were identified involving in several *nad* genes splicing. It would be needed to deeply determine whether and how the rest of P‐type PPR proteins involving in organelles RNA metabolism.

### Disruption of the *OsNBL3* results in enhanced tolerance to salinity stress

As one of the major abiotic factors limiting crop productivity, high salinity stress induces osmotic stress and ionic stress that inhibit water uptake in roots and photosynthesis in shoots (Deinlein *et al*., 2014). Salinity stress causes over‐reduction in the mitochondrial electron transport chain (mtETC), resulting in electron leakage to O_2_ and subsequent production of reactive oxygen species (ROS) O_2_^−^ and H_2_O_2_ (Liberatore, *et al*., 2016). Excess ROS is toxic and leads to metabolic disorders, cell damage and cell death (Liberatore, *et al*., 2016; Miller, *et al*., 2010). The complex I and III of the mtETC are major ROS‐producing sites under abiotic stress (Miller, *et al*., 2010). In plants, mitochondria can bypass the oxidative phosphorylation pathway and transport protons without producing ATP, by depending on alternative NAD(P)H dehydrogenases (NDs) and alternative oxidases (AOXs) to reduce the superoxide produced by the mtETC (Vanlerberghe, 2013). The alternative respiration pathway is considered to be important for preventing over‐reduction in mtETC and for mitigating ROS production (Wanniarachchi *et al*., 2018). Recently, ATP synthase (CotAD_74681) and cytochrome oxidase (CotAD_46197) in mitochondria were found differently expressing in response to salt stress in cotton, suggesting that mitochondria play an essential role in the synthesis of resistance proteins during the process of salt exposure (Peng, *et al*., 2018). Inhibition of mitochondrial complex I enhances high salinity‐stress tolerance in *Arabidopsis thaliana* (Sako, *et al*., 2020). In this study, the *nbl3* mutant and RNAi plants showed enhanced salt tolerance (Figure 4 and Figure S5a,b). The mutation of *OsNBL3* also reduced the activity of complex I and affected mitochondrial morphology with the collapse of cristae with vesicle‐like structures, and elevated reliance on alternative respiratory pathways (Figure 7 and Figure S7). The destruction of the inner mitochondrial membrane underlies the production of mtROS and results in their leakage into the cytoplasm. Moreover, the leakage of mtROS further destroys the intracellular environment and homeostasis, which via feedback, activates the plant’s protective mechanism against salt stress and triggers HR response, as well as growth retardation and pre‐senescence.

Taken together, it is plausible that the mutation of the *OsNBL3* gene is responsible for the *nbl3* phenotype as the fully functioning gene normally participates in the splicing of *nad5* introns. Disruption of *OsNBL3* leads to cell death and enhanced disease resistance and salt tolerance. Our results would provide an insight to explain the mechanisms underlying plant defence against biotic and abiotic stresses.

## Methods

### Plant materials and growth conditions

The *nbl3* mutant was identified by screening a T‐DNA insertion population of the rice cultivar Aichiasahi (*Oryza sativa* ‘Geng’ [japonica]). The *nbl3* mutant and wild‐type plants were grown in the experimental field at China Agricultural University in Beijing, China, or in growth rooms maintained at 28°C during the day, with a 12‐h light/12‐h dark photoperiod and 70% humidity.

### Pathogen inoculations and salt treatments

Rice seedlings were inoculated with the *Magnaporthe oryzae* isolate H535 using the punch inoculation method (Fang *et al*., 2018). In brief, the detached leaves of five‐leaf‐stage rice seedlings were wound‐inoculated with 10 μL of spore suspension (2 × 10^5^ spores/mL) supplemented with 0.025% Tween 20. Moreover, intact leaves were spray‐inoculated with H535 spore solution (1 × 10^6^ spores/mL) containing 0.025% Tween 20. The inoculated leaves were then transferred to a chamber at 28°C under 100% humidity and a 12‐h dark/12‐h light cycle. The length of the resulting lesions was measured at 96 h post‐inoculation (hpi). To evaluate rice bacterial blight disease resistance, the wild type and *nbl3* mutant were inoculated with the *Xanthomonas oryzae* pv. *oryzae* (*Xoo*) strain PXO99 using the leaf‐clipping method at 60 days after sowing (DAS). The overnight liquid culture of the bacterium was collected and adjusted to an optical density (OD) = 0.8 using deionized water. The distal tip (approximately 3 cm) of the flag leaves was removed using scissors and then dipped into the bacterial suspension. Five individual plants and three tillers per plant were inoculated with PXO99, and these plants were grown in a glasshouse. The lesion length was measured at 14 days post‐inoculation (dpi).

For the salt treatments, seeds of the *nbl3* and wild‐type plants were treated with sodium hypochlorite solution (1%) to accelerate germination. 10‐day‐old seedlings grown in normal conditions (28°C and photoperiod of 12‐h light/12‐h dark) were continuously irrigated with 100 mm or 150 mm NaCl, while plants irrigated only with water were used as the control. The whole plants were photographed after thirty days of irrigation. During the irrigation with NaCl, wild‐type leaves were sampled at different time points to conduct expression analysis of *OsNBL3* by real‐time quantitative polymerase chain reaction (RT‐qPCR). In addition, germinated seeds were placed on agar plates containing 0, 100, 150 or 200 mm NaCl, and grown in a greenhouse (28°C and photoperiod of 12‐h light/12‐h dark). The seedlings under each treatment were photographed, and plant height and fresh weight were measured at 7 d. Also, roots of the seedlings under 100 mm NaCl were photographed, and the root lengths were measured at 2, 3, 4 and 5 d.

### Site finding thermal asymmetric interlaced (TAIL)‐PCR for *OsNBL3* cloning

SiteFinding TAIL‐PCR procedures were used to isolate the sequences flanking the T‐DNA using a previously described method (Tan *et al*., 2005). The products of tertiary SiteFinding TAIL‐PCR were sequenced and used to search against the rice genome database (http://rice.plantbiology.msu.edu/) to obtain the T‐DNA insertion site. Specific primers, L3, R3, JD‐F and JD‐R, were used to reconfirm the T‐DNA insertion site. The sequences of all of the primers used in the study are listed in Table S2.

### Transmission electron microscopy

Leaf sections of the *nbl3* and wild type were sampled from plants at 60 DAS, and immersed in 2.5% glutaraldehyde in a phosphate buffer at room temperature for 48 h and then maintained at 4°C overnight. Subsequently, the samples were washed and incubated in 1% OsO_4_ at 4°C for 12 h. After dehydration in a gradient ethanol series, the samples were embedded in Spurr’s resin prior to ultrathin sectioning. Sections were stained with uranyl acetate and examined with a Hitachi‐H7500 transmission electron microscope.

### Histochemical assays

The second leaves of the *nbl3* and wild type were used in histochemical assays at 60 DAS. A lactic acid‐phenol‐trypan blue solution was used to evaluate cell death, and tetranitroblue tetrazolium chloride (NBT) solution and 3,3′‐diaminobenzidine (DAB) solution were used to evaluate H_2_O_2_ accumulation. Staining was performed using previously described methods (Wang *et al*., 2017c). Briefly, leaves of the *nbl3* and wild‐type plants were immersed in Trypan blue solution (0.25% Trypan blue, 25% lactic acid, 23% water‐saturated phenol and 25% glycerol) in a boiling water bath for 20 min, cooled all night and supplemented with chloral hydrate (0.25%) for 30 h. Leaves were also immersed in DAB solution (1 mg/mL DAB and 10 mm Na_2_HPO_4_; pH 3.8) or NBT solution (0.5 mg/mL NBT and 10 mm K_2_HPO_4_; pH 7.8) in the dark for 16 h at room temperature. The staining leaves were transferred into 95% ethanol to decolourize.

### Subcellular localization

To perform transient expression analysis in rice protoplasts and *Nicotiana benthamiana* leaf epidermal cells, the full‐length coding sequence of *OsNBL3* without the stop codon and with a total length of 300 bp starting from ATG was amplified using the primer set OsNBL3‐GFP‐F/R or 3N‐GFP‐F/R, respectively. The PCR product was fused in frame with green fluorescent protein (GFP) in the pCAMBIA1301 plasmid, to generate 35S:OsNBL3‐GFP or 35S:3N‐GFP fusion constructs, respectively. The expression vector was introduced into rice protoplasts following previously described methods (Zhao *et al*., 2020), and the transfected protoplasts were incubated at 28 °C in the dark. The fluorescent GFP signal was examined and photographed using a laser confocal scanning microscope (Leica TCS SP8) after 16 h. The mitochondria dye Mito‐Tracker Red (Invitrogen) was incubated at 37°C in a suspension containing the rice protoplasts 15 min before imaging (Shyu *et al*., 2008). Additionally, pCXSN‐COX4‐RFP (Chen et al., 2019) as Mito‐Marker constructs and 3N‐GFP or OsNBL3‐GFP fusion constructs were introduced into the *Agrobacterium tumefaciens* strain EHA105. The constructs were then transformed via *A. tumefaciens* into *Nicotiana benthamiana* leaves together using previously described methods (Shyu *et al*., 2008). Fluorescence was detected 36 h after infiltration. The primers used are listed in Table S2.

### RNA isolation and RT‐qPCR analysis

For the expression analysis of defence marker genes and AOX or ND genes, the leaves of five‐leaf‐stage wild‐type and *nbl3* plants were used. For the expression pattern analysis, the leaves from the top to bottom, the sheath, stem nodes, panicles and roots of the wild type were used. Total RNA was extracted using the KK Fast Plant Total RNA Kit (Beijing Zoman Biotechnology), and cDNA synthesis was performed using the HiScript II 1st Strand cDNA Synthesis Kit (+gDNA wiper) according to manufacturers’ instructions. RT‐qPCR was performed using 2x RealStar Green Power Mixture with ROX II and ABI QuantStudio 6 Flex PCR program, with the rice actin gene (*LOC_Os03g50885*) used as an internal control. Primers used for the RT‐qPCR analyses are listed in Table S2.

### Measurement of phytohormones

For measurement, the intensity of JA, ABA and SA, ~1 g (fresh weight) rice seedlings were used to extract phytohormones and their metabolites, followed by Cao *et al*. (2016). The resulting sample solution was injected into the LC–MS/MS (1260‐6520 LC‐QTOF, Agilent) for further analysis. Phytohormones and their metabolites were separated on a ZORBAX SB‐C18 column (2.1*100 mm, 3.5 µm, Agilent). The mobile phase consisted of water with 0.1% formic acid (solvent A) and ACN with 0.1% formic acid (solvent B). The gradient is started with 5% B and kept for 2 min, then increased to 25% B in 8 min, to 70% in 30 min, to 95% B in 3 min and kept for 7 min. The flow rate was 0.25 mL/min. The parameters for MS are as follows: ESI source, negative mode, capillary voltage = 3000V, gas temperature = 340°C. Data acquisition and analysis were performed using MassHunter software (Agilent). The standard solution of JA, ABA and SA were used for identification. *m/z* of 209.1182, 138.0317 and 264.1362 were used for identification of JA, SA and ABA, respectively. The intensity of JA, ABA and SA in each sample was log_10_‐transformed and then compared. All the samples were analysed with three biological replicates.

### Circularized RNA reverse transcription PCR (CR‐RT‐PCR)

Following the T4 RNA Ligase I (New England Biolabs) guide, five µg of total RNA from rice leaves at 30 DAS were circularized. First‐strand cDNA was synthesized using Prime Script II RTase (TaKaRa) with specific primers (Table S2). The resulting cDNAs were amplified using primers specific to each target gene (Table S2). Each circular RT‐PCR fragment was cloned into the pCloneEZ‐Blunt TOPO Cloning Kit (Clone Smarter) with ten monoclonals for each target gene sequence.

### Vector construction and rice transformation

For the genetic overexpression and RNAi tests, the *OsNBL3* coding sequence was amplified using cDNA templates derived from the rice cultivar Aichiasahi. PCR products were cloned into the binary vector pCAMBIA1301 under the control of the maize ubiquitin promoter to generate fused pUbi:3Flag+OsNBL3 constructs. Bases 238 to 797 of the cDNA fragment of *OsNBL3* were selected and used for RNAi vector construction. The 386 bp intron of *OsNBL3* was used as a linker. The fragments of *OsNBL3* were fused into pUbi:OsNBL3‐RNAi constructs. The vectors were introduced into *Agrobacterium tumefaciens* strain EHA105 and used to infect wild‐type calli. Primers used in the study are listed in Table S2.

### Blue Native‐PAGE electrophoresis and Complex I activity assays

Crude mitochondria were isolated from germinating seedlings of two days, and 120 µg of mitochondria protein samples were run for BN–PAGE according to Zhang *et al*. (2017). In‐gel NADH dehydrogenase activity assay was performed as described by Meyer *et al*. (2009). Briefly, the gel was washed three times for 5 min with distilled water and incubated in the reaction medium (0.14 mm NADH, 1.22 mm NBT, and 0.1 M Tris‐HCl, pH 7.4). When the dark blue stain was strong enough, the reaction was stopped by transferring the gel to 40% methanol/10% acetic acid (v/v).

## Conflict of interest

The authors declare that they have no conflicts of interest.

## Author contributions

WS Zhao designed the research; TC Qiu, XS Zhao, HJ Feng and LL Qi performed the experiments; WS Zhao, TC Qiu, J Yang and YL Peng discussed the results; WS Zhao and TC Qiu wrote the manuscript. The author(s) read and approved the final manuscript.

## Supporting information


**Figure S1** Several agronomic traits of the *nbl3* mutant and wild type (WT) at the pollination stage.
**Figure S2** Molecular cloning of the *OsNBL3*.
**Figure S3** Phenotypes of the whole plants of the wild type, *nbl3* mutant and three representative RNAi lines, and the expression of *OssNBL3* in these plants.
**Figure S4** RT‐qPCR analyses of two defence‐related genes in the seedling of the wild type, *nbl3* mutant and three represent *RNAi* lines.
**Figure S5** Disruption of *OsNBL3* results in enhanced salt tolerance in seedlings.
**Figure S6** Circular RT‐PCR analysis of seventeen mitochondrial genes.
**Figure S7** Detailed mitochondrial morphology and expression of alternative respiratory pathway genes in the *nbl3* and wild type.
**Table S1** Genetic analysis of the *nbl3* mutant.
**Table S2** List of primers and their uses.
