# Supplementary material for: OsNBL3, a mitochondrion‐localized pentatricopeptide repeat protein, is involved in splicing nad5 intron 4 and its disruption causes lesion mimic phenotype with enhanced resistance to biotic and abiotic stresses
Source: Plant Biotechnol J. 2021 Jul 17;19(11):2277–90. doi: 10.1111/pbi.13659 (PMC8541779; doi:10.1111/pbi.13659)
Supplement: Supplementary file 1 — Figure S1 Several agronomic traits of the nbl3 mutant and wild type (WT) at the pollination stage. Figure S2 Molecular cloning of the OsNBL3. Figure S3 Phenotypes of the whole plants of the wild type, nbl3 mutant and three representative RNAi lines, and the expression of OssNBL3 in these plants. Figure S4 RT‐qPCR analyses of two defence‐related genes in the seedling of the wild type, nbl3 mutant and three represent RNAi lines. Figure S5 Disruption of OsNBL3 results in enhanced salt tolerance in seedlings. Figure S6 Circular RT‐PCR analysis of seventeen mitochondrial genes. Figure S7 Detailed mitochondrial morphology and expression of alternative respiratory pathway genes in the nbl3 and wild type. Table S1 Genetic analysis of the nbl3 mutant. Table S2 List of primers and their uses. [file PBI-19-2277-s001.docx]

**Supporting Information**

OsNBL3, a mitochondrion-localized pentatricopeptide repeat protein, is involved in splicing *nad5* intron 4 and its disruption causes lesion mimic phenotype with enhanced resistance to biotic and abiotic stresses

Tiancheng Qiu, Xiaosheng Zhao, Huijing Feng, Linlu Qi, Jun Yang, You-Liang Peng and Wensheng Zhao

State Key Laboratory of Agrobiotechnology, MOA Key Lab of Pest Monitoring and Green Management, Department of Plant Pathology, China Agricultural University, Beijing 100193, People’s Republic of China.

Correspondence

Wensheng Zhao

Email: [mppzhaws@cau.edu.cn](mailto:mppzhaws@cau.edu.cn)

**Figure S1** Several agronomic traits of the *nbl3* mutant and wild type (WT) at the pollination stage.

**Figure S2** Molecular cloning of the *OsNBL3.*

**Figure S3** Phenotypes of the whole plants of the wild type, *nbl3* mutant and three represent RNAi lines, and expression of *OssNBL3* in these plants.

**Figure S4** RT-qPCR analyses of two defense-related genes in the seedling of the wild type, *nbl3* mutant and three represent *RNAi* lines*.*

**Figure S5** Disruption of *OsNBL3* results in enhanced salt tolerance in seedlings.

**Figure S6** Circular RT-PCR analysis of seventeen mitochondrial genes.

**Figure S7** Detailed mitochondrial morphology and expression of alternative respiratory pathway genes.

**Table S1** Genetic analysis of the *nbl3* mutant.

**Table S2** List of primers and their uses.


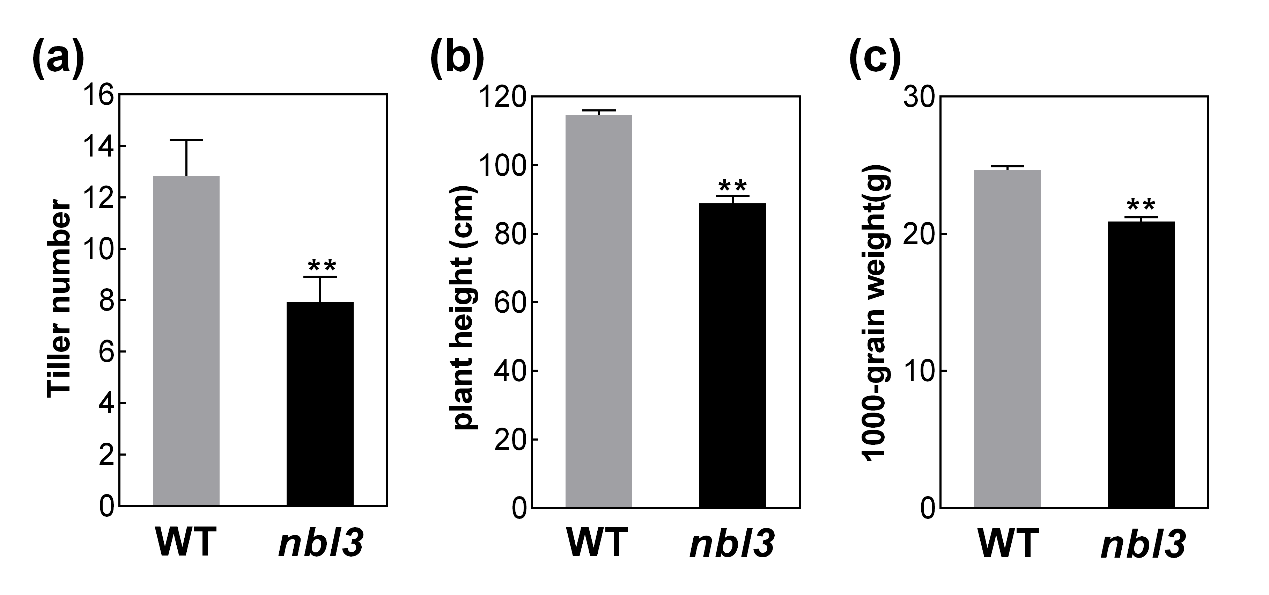


**Figure S1 Several agronomic traits of the *nbl3* mutant and wild type (WT) at the pollination stage.** The *nbl3* mutant decrease tillers number (a), plant height (b) 1000-grain weight (c) compared with WT plants. Data were shown as means ± SD, n=15, (***P* < 0.01; Student’s *t* test).
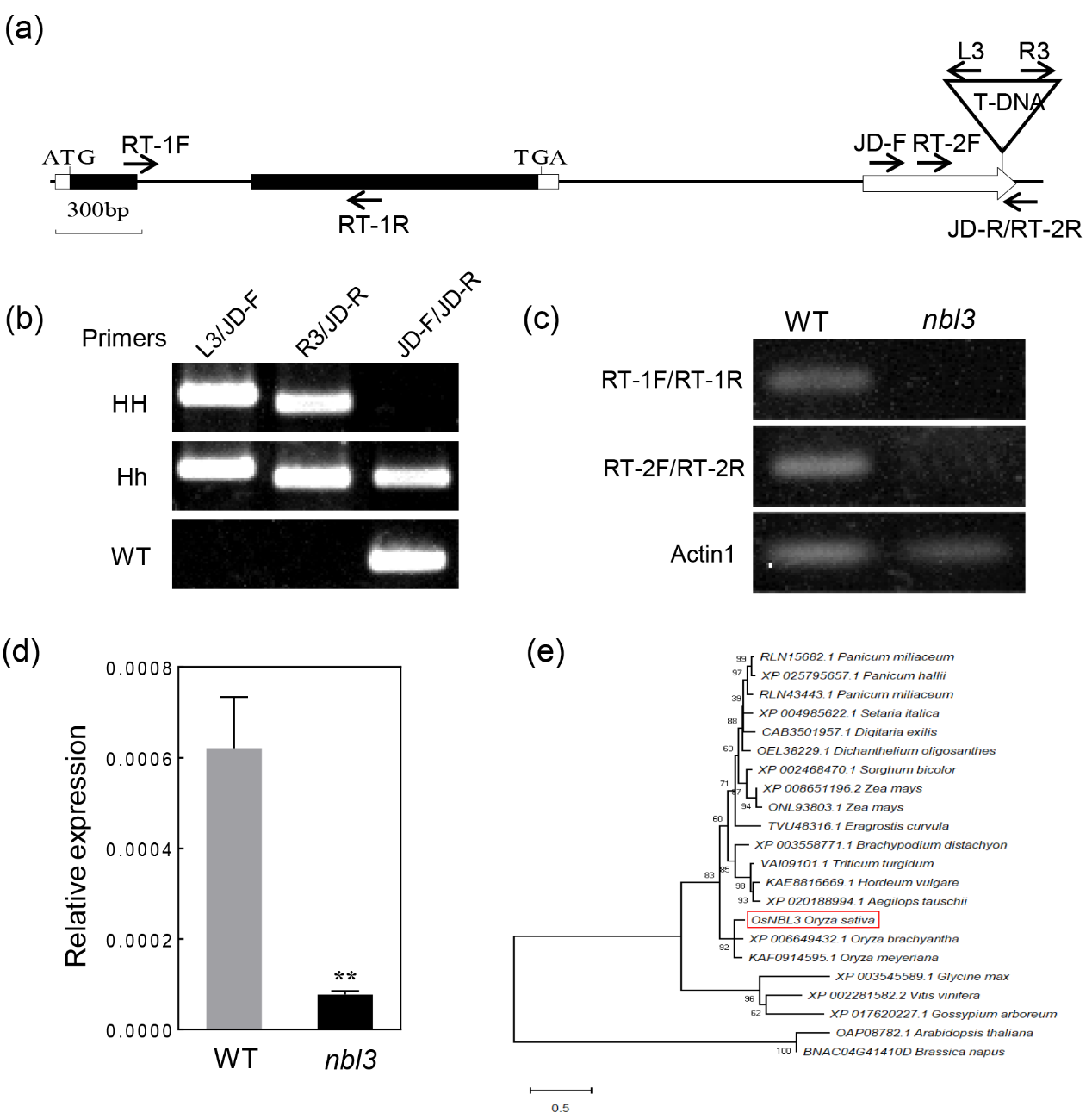


**Figure S2 Molecular cloning of the *OsNBL3.*** (a) A schematic diagram showing the T-DNA insertion event in the *nbl3* mutant. The triangle indicates the T-DNA insertion site in the *nbl3* mutant. Black boxes indicate exons, lines indicate introns, and white boxes indicate the untranslated regions. Primers used to confirm the insertion event and conduct expression analysis were marked. (b) Confirmation of the T-DNA insertion event by PCR using the specific primers (L3/JD-F, R3/JD-R and JD-F/JD-R); HH and Hh indicate homozygous and heterozygous mutant plants, respectively. (c) and (d) Expression analysis of *OsNBL3* in the wild type(WT) and *nbl3* plants by semi quantitative RT-PCR and RT-qPCR, respectively. Rice gene *Actin1*(*LOC_Os07g38730*) was used as an internal control, data were shown as means ± SD, n=3, (***P* < 0.01; Student’s *t* test). (e)A neighbour-joining tree of OsNBL3 and its homologues. The tree was constructed using MEGA X and was bootstrapped with 1000 replicates. The proteins are named according to their NCBI accession numbers. OsNBL3 is indicated by the red frame.


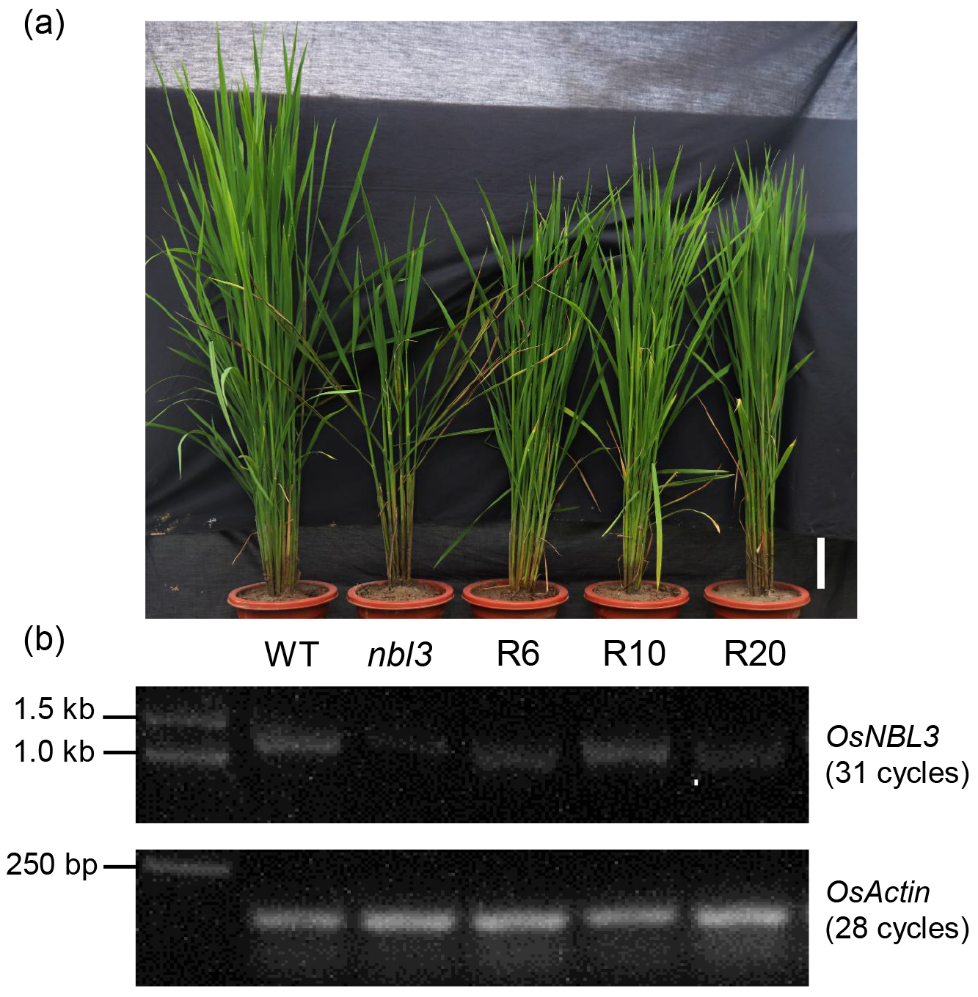


**Figure S3 Phenotypes of the whole plants of the wild type, *nbl3* mutant and three represent RNAi lines, and expression of *OssNBL3* in these plants.** (a) Morphology of the whole plants of the wild type, *nbl3* mutant and three represent RNAi lines at the pollination stage in the paddy field. Scale bars=10.0 cm. (b) Semi quantitative RT-PCR analyses using RT-F and RT-R as primers to evaluate intact mRNA level of the *OsNBL3* in these plants*.* The *OsActin* gene (*LOC_Os03g50885*) was used as an internal control.





**Figure S4 RT-qPCR analyses of two defense-related genes in the seedling of the wild type, *nbl3* mutant and three represent RNAi lines*.*** The *OsActin* gene (*LOC_Os03g50885*) was used as an internal control. Data were shown as means ± SD, n=3, (***P* < 0.01; Student’s *t* test).


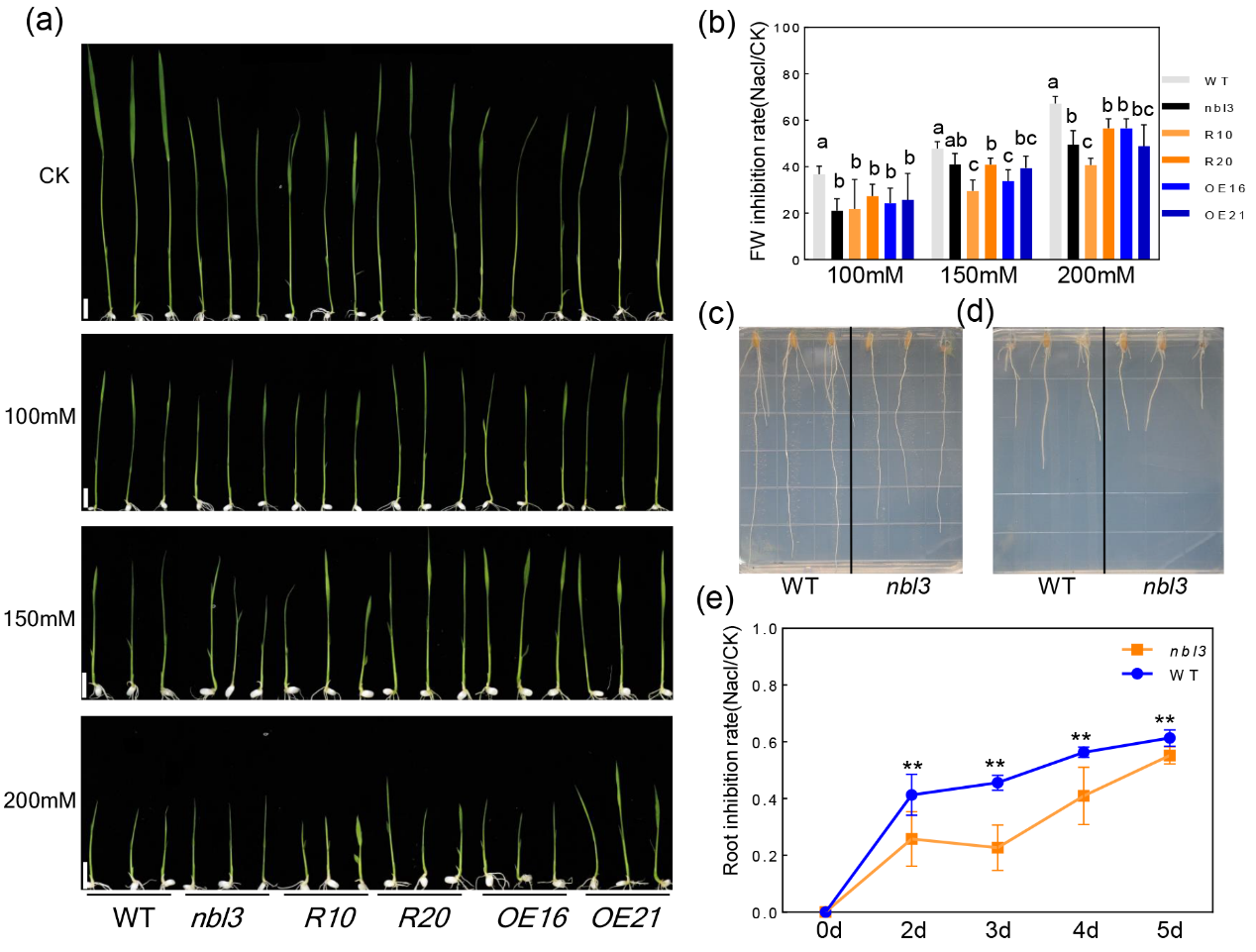


**Figure S5 Disruption of *OsNBL3* results in enhanced salt tolerance in seedlings.** (a) Morphology of seven-day-old seedlings the wild type, *nbl3*, RNAi-10, -20 and OE-16, -21. Grown on 1/2 MS medium containing 0 mM (CK), 100 mM ,150 mM and 200 mM NaCl, respectively. Scale Bar =1 cm. (b) The inhibition rate of fresh weight (FW) of the seedlings from (a). Data were shown as means ± SD, n=5, (Values with same superscript letters are of no significant difference (P> 0.05), those with different letters are of significant or extreme difference (P< 0.05). (c)and (d) Effects of salt treatment on roots growth of the wild type and the *nbl3* mutant. Fifteen germinated seeds were planted on agar medium containing 100 mM Nacl for 5 days, respectively (d). Those growing on agar medium were used as control (c). (e) The inhibition rate of roots growth of the wild type and the *nbl3* mutant by treatment with 100 mM NaCl. Roots length were measured at different time points. Data were shown as means ± SD, n=15, (***P* < 0.01; Student’s *t* test).


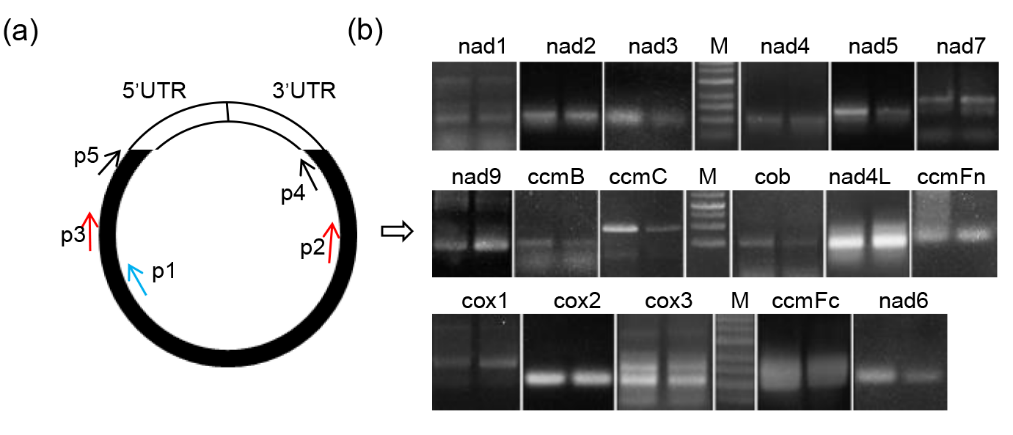


**Figure S6 Circular RT-PCR analysis of seventeen mitochondrial genes.** (a) A schematic diagram of circular RT-PCR procedures. The blue arrow indicates the circularized amplification primer P1; the red arrows indicate the first round nested amplification primers P2 and P3; the black arrow indicates the second round of nested amplification primers P4 and P5; the black box indicates CDS, and white boxes indicate the untranslated regions. (b) Electrophoresis of PCR products amplified using corresponding P4 and P5 as primers. M, DNA ladder.


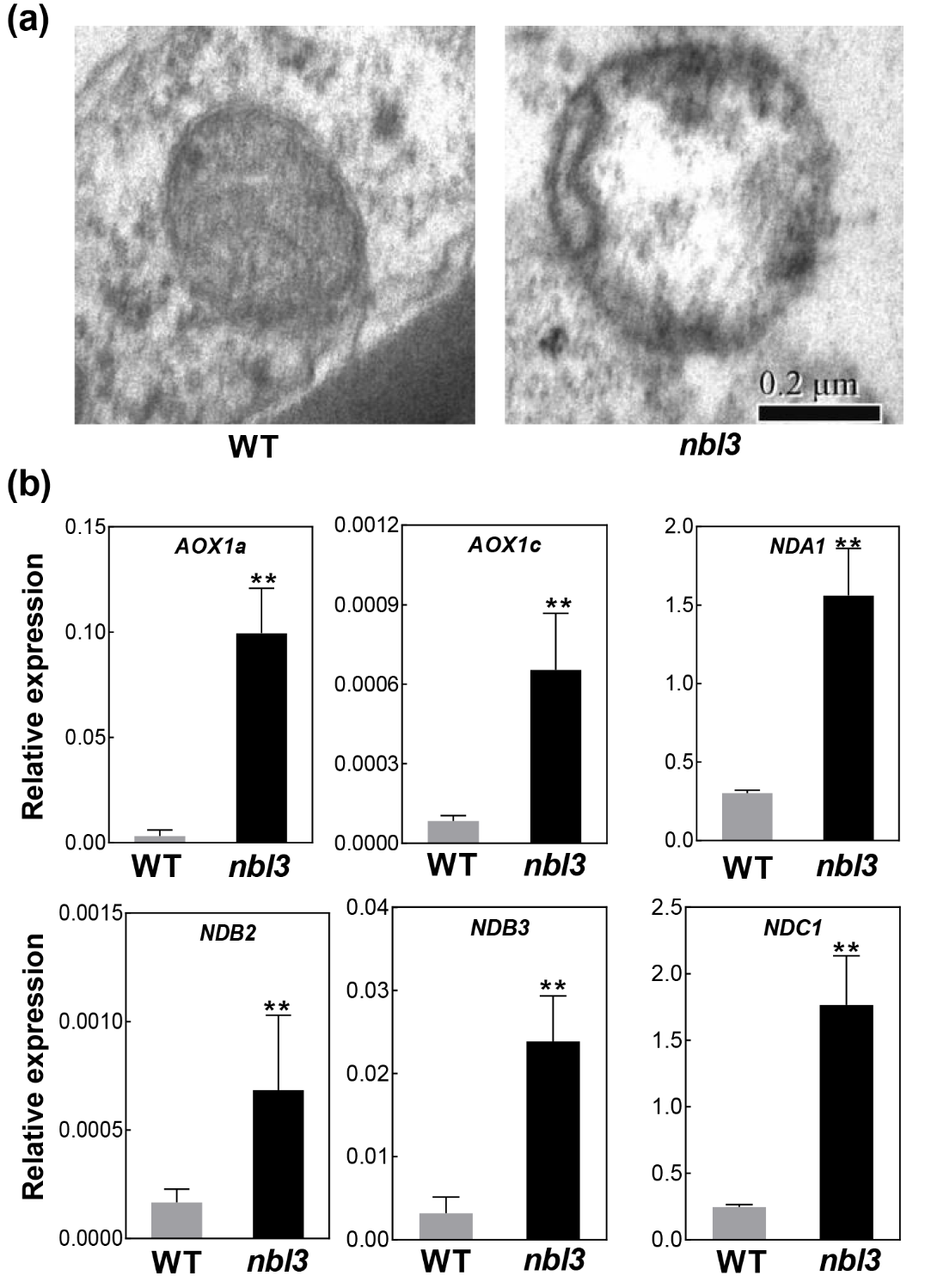


**Figure S7 Detailed mitochondrial morphology and expression of alternative respiratory pathway genes in the *nbl3* and wild type.** (a) Enlarged transmission electron microscope images of mitochondria in leaves of the wild type (WT) and the *nbl3* mutant. Bar = 0.2 μm. (b) Real-time quantitative polymerase chain reaction (RT-qPCR) analyses of alternative oxidase (AOX) genes and NADH dehydrogenase (ND) genes. The *rrn18* (*LOC_Os03g42530*) gene was used as an internal control. Data are shown as means ± SD, n = 3 (***P* < 0.01; Student’s *t* test).

**Table S1** Genetic analysis of the *nbl3* mutant.

| Class | T1  plants | T2 HygB resistance  Homozygous plants | T2 HygB resistance  Separation plants | T2 HygB susceptible plants |
| --- | --- | --- | --- | --- |
| Mutant phenotype | 24 | 24 | 0 | 0 |
| WT phenotype | 89 | 0 | 59 | 30 |
| total | 113 | 24 | 59 | 30 |

**Table S2** List of primers and their uses.

| Use | Primer name | Sequence (5’-3’) | Reference |
| --- | --- | --- | --- |
| Cloning of *OsNBL3* | JD-F | GCATGTCTCAAGGTGGAACTGT | This study |
|  | JD-R | TGTGCACAATCAACCTACTTCAT |  |
|  | L3 | GATGCCGACCGGATCTGTCGATC |  |
|  | R3 | CTGTTGCCGGTCTTGCGATGAT |  |
|  | RT-1F | CTAAGGCAAACCTCGTCTTG |  |
|  | RT-1R | GAGTAAATCCTGCATCCTTTGAT |  |
|  | RT-2F | GGACGATGAATCTATACATGAACACA |  |
|  | RT-2R | ATGTGCACAATCAACCTACTTCAT |  |
|  | RT-F | TATATCCACGCGATTCACTGG |  |
|  | RT-R | TCATGCAAACCCAGCTTCCC |  |
| RT-qPCR analysis for defense-related genes | OsPR1b-F | TCGTATGCTATGCTACGTGTTT |  |
|  | OsPR1b-R | CACTAAGCAAATACGGCTGACA |  |
|  | OsPR2-F | CTGGCATTGGTCCTTGGAGTT |  |
|  | OsPR2-R | CGATGCCGTTGGACTTGTAG |  |
|  | OsPR3-F | CCTATTGCATGATCGTTCGAT |  |
|  | OsPR3-R | GCCTGTAGCAGTTAAAGCAATTG |  |
|  | OsPR5-F | CCACGTGTGCAATTGTTTAATC |  |
|  | OsPR5-R | ACTCGGACGCTTTCATTTGA |  |
|  | OsPR8-F | TTCATCTGGTCAGCGGATAGC |  |
|  | OsPR8-R | TATCACGACCGTTCGATGGA |  |
|  | OsPR10-F | CCTGCCGAATACGCCTAAGA |  |
|  | OsPR10-R | CTCAAACGCCACGAGAATTT |  |
|  | OsWRKY45-F | AGCAATCGTCCGGGAATTC |  |
|  | OsWRKY45-R | GAAGTAGGCCTTTGGGTGCTT |  |
|  | OsWRKY62-F | TGAAGGATGGGTACCAATGGA |  |
|  | OsWRKY62-R | CACATCTTTGGAGCTTCTTCTTGA |  |
| RT-qPCR analysis for salt associated genes | OsHAK1-F | GTTGATGATGCTGATGTTGGAAG |  |
|  | OsHAK1-R | CCAACACTTTCAGCTGAAAC |  |
|  | OsLEA3-F | GGCGAGTGAGCAGGTGAAGAG |  |
|  | OsLEA3-R | GCGGTGGCAGAGGTGTCC |  |
|  | OsNAC22-F | CTTCAACATCATCGGCACCC |  |
|  | OsNAC22-R | AGAAGTACCACTCCCTCTCCC |  |
|  | OsNAP-F | AAGGGCACCAAGACCAACTG |  |
|  | OsNAP-R | TCTTGTAGATCCGGCACAGC |  |
|  | OsMYB4-F | ACCATCATCCATCTCCACGAG |  |
|  | OsMYB4-R | TTGATCTCGTTGTCCGTCCT |  |
| construction of expression vector | OsNBL3-OE-F | AATACTAGTTATATCCACGCGATTCACTGG |  |
|  | OsNBL3-OE-R | ATAACGCGTATGTGCACAATCAACCTACTTCAT |  |
|  | OsNBL3-RNAi-F1 | GGCCTTAATTAATGAGTGAGACACCCTTC |  |
|  | OsNBL3-RNAi-R1 | ATAACGCGTCCATCGCTTACAAGTTCC |  |
|  | OsNBL3-RNAi-F2 | CGGGTTAATTAATGGAATGGAAGCTAGAG |  |
|  | OsNBL3-RNAi-R2 | ATCGTTTAAACCCATCGCTTACAAGTTCC |  |
|  | OsNBL3-GFP-F | ATAGAGCTCTCACCGATGGCGATC |  |
|  | OsNBL3-GFP-R | ATAGTCGACTGCAAACCCAGCTTCC |  |
|  | 3N-GFP-F | ATAGAGCTCATGGCGATCGC |  |
|  | 3N-GFP-R | ATAGTCGACGTAGGGCTCACAAT |  |
| RT-qPCR analysis for alternative respairy pathway genes | NDA1F | ATAAGCATCTGGGGAGCATG | (Toda, et al., 2012) |
|  | NDA1R | AAATACGGCTAATGTCACGG |  |
|  | NDB2F | AAGGTACCCACAGGTTGAGT |  |
|  | NDB2R | GTTGAAGCAGTTTGCCAGAT |  |
|  | NDB3F | AGCAGTTGGAGCTAGGTCTA |  |
|  | NDB3R | CAAAATCGTGCAACTCTGCG |  |
|  | NDC1F | TGTGGGATTGGGTTACTCTG |  |
|  | NDC1R | GCTACTTGAATCCTCAGATG |  |
|  | AOX1aF | GTCTACTGCCGAGGATTTGCATCAC |  |
|  | AOX1aR | CCGATACAGCTAAAGAGCTCTC |  |
|  | AOX1cF | AACACGAGTTGCTGTTGATCTGTGC |  |
|  | AOX1cR | TTCCATGCACACATGAATGCGGATC |  |
| CR-RT-PCR analysis for mitochondrial genes | ccmB1 | CAATTCGAGTGTACCAGATTC | This study |
|  | ccmB-1F | TGACGACGATATACGAGAGG |  |
|  | ccmB-1R | CCTCTATAGAAACAGAAGGGT |  |
|  | ccmB-2F | AATTGTCAAGAAAGAGTCGTC |  |
|  | ccmB-2R | CTCGATTAGTTCACAAGATTG |  |
|  | ccmC1 | CGCAGGAACATGTACATACG |  |
|  | ccmC-1F | TAGCCATCGCTGTTAAGAAC |  |
|  | ccmC-1R | GAAACACGTCTTCCTATTCC |  |
|  | ccmC-2F | GTTGTAATAACGAAACTGACAT |  |
|  | ccmC-2R | CGAGAAGGAATACCACTAAA |  |
|  | ccmFn1 | AGACCATGCCCCTGAGATTT |  |
|  | ccmFn-1F | GGAAGAAGAATGCACCAAAA |  |
|  | ccmFn-1R | CTTATTGCTGGGCTGGTT |  |
|  | ccmFn-2F | AAAAGACCCGGAAATAACAA |  |
|  | ccmFn-2R | ATTTCGTCCCGTCCGTAG |  |
|  | ccmFc1 | CACTCCACTTTTTGCTCTGT |  |
|  | ccmFc-1F | GGGAACATCTCTACTGACGA |  |
|  | ccmFc-1R | CGGATAGGATTTCTGTTGG |  |
|  | ccmFc-2F | GAAGTTCTGTAGTTGGACCAT |  |
|  | ccmFc-2R | AGGATAAGTTGCATTGGAAT |  |
|  | cox3a | TAGAACATCCCGCCACCATA |  |
|  | cox3-1F | AAAGCTCCGAGTGAACCC |  |
|  | cox3-1R | ATCTGACCAAGAAGCATCAC |  |
|  | cox3-2F | TATGTAAACCCCCCTTCACC |  |
|  | cox3-2R | ATTGGTGGGGAGGTATATGA |  |
|  | cox2a | GCGAACCAACATCCGTGATA |  |
|  | cox2-1F | ATCATAGGTGTTGCAGCGT |  |
|  | cox2-1R | AATCATGCCTTTACGCCTAT |  |
|  | cox2-2F | GAGGAATCGACATTCTAATGAA |  |
|  | cox2-2R | TTAATCCTCCAAACCAACTAA |  |
|  | cox1a | TACCAAATCCACCTATCATCGC |  |
|  | cox1-1F | GAGAAGCATGTGCCCATCA |  |
|  | cox1-1R | AACAGAATCCAACCACACTAG |  |
|  | cox1-2F | CCATCGGACCAGATTTGT |  |
|  | cox1-2R | CTTCCAGCTATCAAAAAGACAA |  |
|  | cob1 | AAAACGCCAGTCACTATCTG |  |
|  | cob-1F | CTAAGATTGCTCGGAGTTGG |  |
|  | cob-1R | TTGTTCTTTGCCATAACGC |  |
|  | cob-2F | ATCGTTGGTTCCTTATAGTCAT |  |
|  | cob-2R | CACCGGATCAGTCTCTTAG |  |
|  | Nad1a | GAACGATCCCACTACATCAG |  |
|  | Nad1-1F | CTTTACGTTCAGCTAGCACT |  |
|  | Nad1-1R | ATATGGGTCCGTGCAGCATT |  |
|  | Nad1-2F | GCTGGAACAGCTATGTACGT |  |
|  | Nad1-2R | CCTTTCGATGGCTCCCTTAA |  |
|  | Nad2a | CAGAAGCAAGGTTATTAGAA |  |
|  | Nad2-1F | TACAACTCCATGAATGAGC |  |
|  | Nad2-1R | GGATCGTGACAAGTCGTTAC |  |
|  | Nad2-2F | CCGCTAAAAAAAGATTGAACAT |  |
|  | Nad2-2R | GGCACTCAGTTCATATCTGTAA |  |
|  | Nad3a | CAAAGAAACTAGCGGACTGA |  |
|  | Nad3-1F | TCCGTATTCCCTGAAGAAC |  |
|  | Nad3-1R | CCATGATGGCCTTTTTATTG |  |
|  | Nad3-2F | ACACCAAAAAAAATGTTGTCC |  |
|  | Nad3-2R | TGCTTCGGATCGGGAGTAA |  |
|  | Nad4a | GTAATAAGAGAAACGCACAG |  |
|  | Nad4-1F | GGAATGAAAAGAAGAGTAATGC |  |
|  | Nad4-1R | CTTGTTGGAGTTGTTCGGAT |  |
|  | Nad4-2F | GCATTCACAGAAATGTTCTAAC |  |
|  | Nad4-2R | GCAACATGGCAAATTTCATTG |  |
|  | Nad5a | TCTGAGATAGCAAGCACTAG |  |
|  | Nad5-1F | CAGATCCTAGAAAACGTCCG |  |
|  | Nad5-1R | ATGTGGGACTCTCTATCTTC |  |
|  | Nad5-2F | GCAAAAAGACAATAAGTAGATAC |  |
|  | Nad5-2R | CTTATTAATAAGTCAAGTCAAGAA |  |
|  | Nad6a | AACTACTGGGGAGATCATAG |  |
|  | Nad6-1F | GACTAGGATGGGAAACAAAAC |  |
|  | Nad6-1R | AGACAGGATGTATTCCGACG |  |
|  | Nad6-2F | CGACAAAACTGAAAGTATCAT |  |
|  | Nad6-2R | CAGACCAAATCACGATCTAC |  |
|  | Nad7a | TGTTCTTGGGCCATCGTAG |  |
|  | Nad7-1F | CTGATCGTGAAACACCATGA |  |
|  | Nad7-1R | CACAAGGACTCGATTCTATGT |  |
|  | Nad7-2F | CCCGTTCCTAGTCGTCAT |  |
|  | Nad7-2R | GTTTGGAGAGGTAGATAGATAG |  |
|  | Nad9a | GAATGCGTGAGTTATACCGAGT |  |
|  | Nad9-1F | CCCATGTTCCGATCTTTTCA |  |
|  | Nad9-1R | TGATGATCCAGAGAAACGTG |  |
|  | Nad9-2F | TGGAAAATGGATTGGTTATC |  |
|  | Nad9-2R | AACAGCGTAGCGACGGATAA |  |
|  | Nad4L1 | CCCATCATATCATCCAAAGAA |  |
|  | Nad4L-1F | TAAGGAGGATTCCCCGAATA |  |
|  | Nad4L-1R | GCGGAATCTGCTATTGGATT |  |
|  | Nad4L-2F | GTGAAATATTTTATAGGATCCGT |  |
|  | Nad4L-2R | GGACTATTGCTGTCGAATTT |  |
| Reverse transcription PCR analysis for mitochondrial genes | nad1-RT-F | ATGTACATAGCTGTTCCAGCGGAAATA | (Wu, et al., 2019) |
|  | nad1-RT-R | TTAAGGGAGCCATCGAAAGGTGACTGA |  |
|  | nad2-RT-F | ATGTTCAATCTTTTTTTAGCGGTT |  |
|  | nad2-RT-R | TTACAGATATGAACTGAGTGCCAT |  |
|  | nad4-RT-F | ATGTTAGAACATTTCTGTGAATGCTA |  |
|  | nad4-RT-R | TCAATGAAATTTGCCATGTTGCACTA |  |
|  | nad5-RT-F | ATGTATCTACTTATTGTCTTTTTGCCT |  |
|  | nad5-RT-R | TTATTCTTGACTTGACTTATTAATAA |  |
|  | nad7-RT-F | ATGACGACTAGGAACGGGCAAATCA |  |
|  | nad7-RT-R | CTATCTATCTACCTCTCCAAACACA |  |
|  | cox2-RT-F | ATGATTCTTCGTTCATTAGAATGTCGA |  |
|  | cox2-RT-R | TTAGTTGGTTTGGAGGATTAATTGA |  |
|  | rpl2-RT-F | ATGAGACAAAGCATAAAGGGGAGA |  |
|  | rpl2-RT-R | CACTCCTGCCCGAAATCCTGCTTT |  |
|  | rps3-RT-F | TTCGGTAAGACTTGATCTGAATCGTA |  |
|  | rps3-RT-R | CTATATTTCGTACGTTTCGGATATA |  |
|  | ccmFc-RT-F | ATGGTCCAACTACAGAACTTCTTCT |  |
|  | ccmFc-RT-R | ATTCCAATGCAACTTATCCTTTTGGA |  |
|  | atp1-RT-F | ATGGAATTCTCACCCAGAGC |  |
|  | atp1-RT-R | CTAATTAATCTCCTTCGCAG |  |
|  | atp6-RT-F | TTTCGATCACAATCATGTGG |  |
|  | atp6-RT-R | AATTACTCATTTTGATGGAG |  |
|  | atp9-RT-F | GCAAAGTCAAGTCTCCACGA |  |
|  | atp9-RT-R | CAAAGAGAGATATCTACACC |  |
|  | ccmB-RT-F | ATGAGACGACTCTTTCTTGA |  |
|  | ccmB-RT-R | TCAATCTTGTGAACTAATCG |  |
|  | ccmC-RT-F | ATGTCAGTTTCGTTATTACA |  |
|  | ccmC-RT-R | CTAGGTTTTTAGTGGTATTC |  |
|  | ccmFn-RT-F | ATGTCTATAAATGAATTTTC |  |
|  | ccmFn-RT-R | CTACGGACGGGACGAAATCC |  |
|  | cob-RT-F | ATGACTATAAGGAACCAACG |  |
|  | cob-RT-R | CTAAGAGACTGATCCGGTGC |  |
|  | cox1-RT-F | ATGACAAATCTGGTCCGATGGCTCT |  |
|  | cox1-RT-R | CTAGCTTTTTGTCTCTTTGA |  |
|  | cox3-RT-F | TCCTTCTTTACCACTTTAGGGGAT |  |
|  | cox3-RT-R | TCATATACCTCCCCACCAAT |  |
|  | nad3-RT-F | ATGGACAACATTTTTTTTGG |  |
|  | nad3-RT-R | TTACTCCCGATCCGAAGCAC |  |
|  | nad4L-RT-F | ACGGATCCTATAAAATATTT |  |
|  | nad4L-RT-R | TTAACCTTGAATGCAATTTA |  |
|  | nad6-RT-F | ATGATACTTTCAGTTTTGTC |  |
|  | nad6-RT-R | TTAGTAGATCGAGATTTGGT |  |
|  | nad9-RT-F | ATGGATAACCAATCCATTTT |  |
|  | nad9-RT-R | TTATCCGTCGCTACGCTGTT |  |
|  | orf B-RT-F | ATGCCTCAACTTGATAAATTGA |  |
|  | orfB-RT-R | TTAGATTATGCTTCCTTGCC |  |
|  | orfX-RT-F | GCCGAAAATGCATTTATCCT |  |
|  | orfX-RT-R | CTAGTCTACTCCTCTTTTTTCTCGA |  |
|  | rps1-RT-F | ATGTTCTTGGTGGATGCAGG |  |
|  | rps1-RT-R | TCAAGTTCTTGTTTGATCTG |  |
|  | rps2-RT-F | ATGAAAAAGACCAATCAAATCAAACT |  |
|  | rps2-RT-R | CTATTTGGCTAATGCCATCCAAA |  |
|  | rps4-RT-F | ATGCCTGCATTAAGATTTAA |  |
|  | rps4-RT-R | TTATATGTTTTGGCCACGTC |  |
|  | rps7-RT-F | ATGGGGGACTTTGATGGTGA |  |
|  | rps7-RT-R | TTACCACCATCTGAAATGCG |  |
|  | rps12-RT-F | AAAGAGGGATGCCTACAAAAAATCA |  |
|  | rps12-RT-R | AGAGGCATCTTCCATTCATTTCGAT |  |
|  | rps13-RT-F | ATGTCATATATCTCAGGAGC |  |
|  | rps13-RT-R | TCATTTCCGAATTAGCTTGC |  |
|  | rps19-RT-F | ATGCCACGACGATCTATATG |  |
|  | rps19-RT-R | TTACTTTTTCCCCTTTCTGC |  |
|  | rpl3-RT-F | ATGTCATATATCTCAGGAG |  |
|  | rpl3-RT-R | TCATTTCCGAATTAGCTTGC |  |
|  | rpl5-RT-F | ATGTTTCCACTCCATTTTCA |  |
|  | rpl5-RT-R | TTACTTAGTTTCCCCCTCATCTTT |  |
|  | mat-r-RT-F | AGAAAGAAAGAAGGGTCGAAGTT |  |
|  | mat-r-RT-R | CTACTTATTAAACATGTAGTTGAGT |  |
| RT-qPCR anaylsis for *nad*5 splicing | nad1int1-Spliced-F | TGCCATATCTTCGCTAGGTG | (Wu, et al., 2019) |
|  | nad1int1-Unspliced-F | GTGACAGAGGATGTGCTCGT |  |
|  | nad1int1-R | TGAGCTGCAGATCGTAATGC |  |
|  | nad1int2-Spliced-F | GCATTACGATCTGCAGCTCA |  |
|  | nad1int2-Unspliced-F | ACATGCAGGGAAACTTGCAC |  |
|  | nad1int2-R | TGCGCCATGACAATCTCACT |  |
|  | nad1int3-Spliced-F | TGTCTAGCAGAAACTAATCG |  |
|  | nad1int3-Unspliced-F | TGGAAGCTGTATGAGCGGTA |  |
|  | nad1int3-R | GATCATATTGGCATACTCTC |  |
|  | nad1int4-Spliced-F | GAGAGTATGCCAATATGATC |  |
|  | nad1int4-Unspliced-F | GTGATACCACCACCTATCAG |  |
|  | nad1int4-R | CTTGATACTAAACCAGATCG |  |
|  | nad2int1-Spliced-F | TGCTCATTCATGGAGTTGTA |  |
|  | nad2int1-Unspliced-F | TCGCTCATGCTGGAGGTACT |  |
|  | nad2int1-R | GCAATAGTTAGGAGAGGTGC |  |
|  | nad2int2-Spliced-F | GTCTGAATTTTCCACGGAAG |  |
|  | nad2int2-Unspliced-F | ATGCGGTGAGAGTCGCACGTA |  |
|  | nad2int2-R | GAAGTGGGTAGCTCCAGTAG |  |
|  | nad2int3-Spliced-F | CTACTGGAGCTACCCACTTC |  |
|  | nad2int3-Unspliced-F | TCGTTGAGAATAAGTCCTTCC |  |
|  | nad2int3-R | GAACCCTCATAGATATCAGG |  |
|  | nad2int4-Spliced-F | GTGGGAGTAGTGACTAGCGT |  |
|  | nad2int4-Unspliced-F | TCGGAGAGGACTCAGCTGTT |  |
|  | nad2int4-R | GTAACGACTTGTCACGATCC |  |
|  | nad4int1-Spliced-F | GGTTGGTCTGGTATGAGAAG |  |
|  | nad4int1-Unspliced-F | CTACCCACTGGAGCTTCGAC |  |
|  | nad4int1-R | CTGATATGCTGCCTTGATCT |  |
|  | nad4int2-Spliced-F | CATTGCCTACTCCTCAGTAG |  |
|  | nad4int2-Unspliced-F | GGTAACTATCTTGTACGGTTCG |  |
|  | nad4int2-R | AGTCGAGTCTTATGTCGGTC |  |
|  | nad4int3-Spliced-F | CTCCGATCTAAATGGCAGAG |  |
|  | nad4int3-Unspliced-F | GTGAGCCTAGTGATAGGAGA |  |
|  | nad4int3-R | GCCATGTTGCACTAAGTTAC |  |
|  | nad5int1-Spliced-F | GCTCCATGGATCTCATCGGA |  |
|  | nad5int1-Unspliced-F | CGCAGATTGGTCTGACTCGT |  |
|  | nad5int1-R | GCTATGCGGATCCTCAGACAT |  |
|  | nad5int2-Spliced-F | GCGAGACAGATTACGATGTC |  |
|  | nad5int2-Unspliced-F | ACAACCTTAACCGCACAAGC |  |
|  | nad5int2-R | TACCTAAACCAATCATCATAT |  |
|  | nad5int3-Spliced-F | ATATGATGATTGGTTTAGGTA |  |
|  | nad5int3-Unspliced-F | GGCTGCGTCTTTACCCCTAT |  |
|  | nad5int3-R | GATCCGCTACGAGATTGACA |  |
|  | nad5int4-Spliced-F | TCTAGTCAGATCGTTCCTGC |  |
|  | nas5int4-Unspliced-F | GAAGCAGTGAGTGGAGATTC |  |
|  | nad5int4-R | CCAAGTAACATTGCAAAGGC |  |
|  | nad7int1-Spliced-F | ACATCCTGCTGCTCATGGTG |  |
|  | nad7int1-Unspliced-F | TTGCGAATGAATGCTAGGCT |  |
|  | nad7int1-R | CGATTAATTTCTCAGTCCCTC |  |
|  | nad7int2-Spliced-F | GAGGGACTGAGAAATTAATCG |  |
|  | nad7int2-Unspliced-F | TGTGATGCAAGTGAACGTGT |  |
|  | nad7int2-R | AGCTCGTAATGGTACCTCAC |  |
|  | nad7int3-Spliced-F | GTACTGTCACTGCACAGCAA |  |
|  | nad7int3-Unspliced-F | AGTGAAGTGGTGGGCCTACC |  |
|  | nad7int3-R | TCTGGTACCTACTGGTACGT |  |
|  | nad7int4-Spliced-F | GTCCTCCATCACGATCTCGA |  |
|  | nad7int4-Unspliced-F | GACTACCGGATCATCGGTCT |  |
|  | nad7int4-R | AACGGTAGGGACGATTACTC |  |
|  | ccmFc-Spliced-F | CACATGGAGGAGTGTGCATC |  |
|  | ccmFc-Unspliced-F | CCTGCAATAATGGTGCGGCTT |  |
|  | ccmFc-R | CATGTAAATGATCGAGACCTCG |  |
|  | cox2-Spliced-F | TGCTATACCATCGTTTGCTCTG |  |
|  | cox2-Unspliced-F | AAGAGTAGGCGTGGAGAGCT |  |
|  | cox2-R | GAGTGACTGTTCATCGGAACTG |  |
|  | rps3-Spliced-F | CTGAATCGTAGTTCAGATCCA |  |
|  | rps3-Unspliced-F | GCTCTGTCACCATTGACTCT |  |
|  | rps3-R | ACACCTACCGAGACGAAAGCC |  |
|  | rpl2-Spliced-F | TGGAGCGTACAAAGTCGATC |  |
|  | rpl2-Unspliced-F | GTACGAGGCTGCTCACGTAC |  |
|  | rpl2-R | CATTCTGGGCAGGTCGCAAG |  |
| Reverse transcription PCR analysis of *nad5* | Nad5-exon-1F | GATCTGAAGGAACCGCTAT | This study |
|  | Nad5-exon-1R | CATCCCAGGAATAATTGG |  |
|  | Nad5-exon-2F | GGTTGGGAAGTGTCTCTGT |  |
|  | Nad5-exon-2R | GCTAGAGAAGCACCTGAAGTA |  |
|  | Nad5-exon-3F | GCTGGTTCTTCGATCAAG |  |
|  | Nad5-exon-3R | AAAGACGATCTAGAATCTACCC |  |
| Internal control | Actin1-F | TACAACGGTTGGCGTCGCAC | This study |
|  | Actin1-R | AACTTGCGCACACGGTCCAG |  |
|  | OsActin-F | ATCACTGCCTTGGCTCCTA |  |
|  | OsActin-R | CATCTGCTGGAATGTGCTG |  |
|  | rrn18F | CCTGGTAGTCCATGCCGTAA | (Toda, et al., 2012) |
|  | rrn18R | CATCCCGTTAAGGACAGGTT |  |
